# Designing a multilayer film via machine learning of scientific literature

**DOI:** 10.1038/s41598-022-05010-7

**Published:** 2022-01-18

**Authors:** Kenta Fukada, Michiko Seyama

**Affiliations:** NTT Device Technology Labs, NTT Corporation, 3-1 Morinosato, Wakamiya, Atsugi, Kanagawa 243-0198 Japan

**Keywords:** Chemistry, Materials science

## Abstract

Scientists who design chemical substances often use materials informatics (MI), a data-driven approach with either computer simulation or artificial intelligence (AI). MI is a valuable technique, but applying it to layered structures is difficult. Most of the proposed computer-aided material search techniques use atomic or molecular simulations, which are limited to small areas. Some AI approaches have planned layered structures, but they require a physical theory or abundant experimental results. There is no universal design tool for multilayer films in MI. Here, we show a multilayer film can be designed through machine learning (ML) of experimental procedures extracted from chemical-coating articles. We converted material names according to International Union of Pure and Applied Chemistry rules and stored them in databases for each fabrication step without any physicochemical theory. Compared with experimental results which depend on authors, experimental protocol is superiority at almost unified and less data loss. Connecting scientific knowledge through ML enables us to predict untrained film structures. This suggests that AI imitates research activity, which is normally inspired by other scientific achievements and can thus be used as a general design technique.

## Introduction

A multilayer functional film is a layered structure formed by stacking materials and integrating multiple functionalities^1–4^. This film resolves various issues simultaneously, but such a problem-solving technique requires innumerable experiments by trial and error. Recently, much attention has been paid to materials informatics (MI), which uses either computer simulation or a neural network (NNW). However, MI is difficult to apply to layered structures because computer simulation methods^5–9^ are limited to a small calculation area at present. Moreover, NNWs^10–15^ also have many obstacles in preparing training data for layered structures. For example, experimental results^16–20^ or specific physical simulations^21–23^ are useful for precisely predicting chemical structures, including those in layered films^20–23^, but they require massive tests in a laboratory or by computer. Meanwhile, various information sources for machine learning (ML) have been studied, with the focus on material databases containing chemical reactions^24–27^, biological inofrmaton^28^, and inorganic crystal structures^29,30^, which have led to proposals for the retrosynthesis of organic molecules or property-fulfilling inorganic compounds. Another recent approach is to utilize published scientific literature^31–36^, which is full of human knowledge. Compared with other data sources, diverse information can easily be gathered with natural language processing tools^36,37^. Scientific knowledge is perpetuated through a series of human research activities, and past literature provides a roadmap to future discoveries^38^. Academic achievements are promising sources of information for MI^39^; however, our survey of databases related to layered structures found that they are lacking in comprehensiveness. Moreover, multiple functionalities are implemented by taking into account trade-offs in some properties, not by solely relying on theory, and previous approaches have difficulty meeting such a complex target^40–42^.

Here, we propose designing multilayer functional films by ML of scientific literature [see Supplementary Information, Section S1 for concept image]. This method uses function-fulfilling substances to estimate the material of each layer in order starting from the substrate. Compared with inference of the whole layered structure at once, it is superior in terms of tolerance to changes in layer number or the order of stacked materials. To estimate multilayer structures from the substrate to the intermediate layer and outermost layer, we developed an inference engine in which multiple NNWs are connected to each other, which we refer to as ‘cascade neural networks’ (CNNWs). We selected NNWs over other technologies, such as support vector machines, for their ease of implementation and high extensibility (see section S2). Moreover, material names were converted with material descriptors characterized by one-hot encoding with International Union of Pure and Applied Chemistry (IUPAC) rules for utilizing functional groups to reflect the bonding force at the interface. This is the first attempt to develop a tool that supports materials researchers and engineers in designing layered films with multi-functionality. To verify this concept, we predicted untrained films selected from published papers, which facilitated the assessment of inference performance, and designed an original functional film through the system as a demonstration. Our approach using scientific literature is a pioneering method of material design for multilayer films.

## Results and discussion

### Data preparation and system overview

**Figure. 1:**
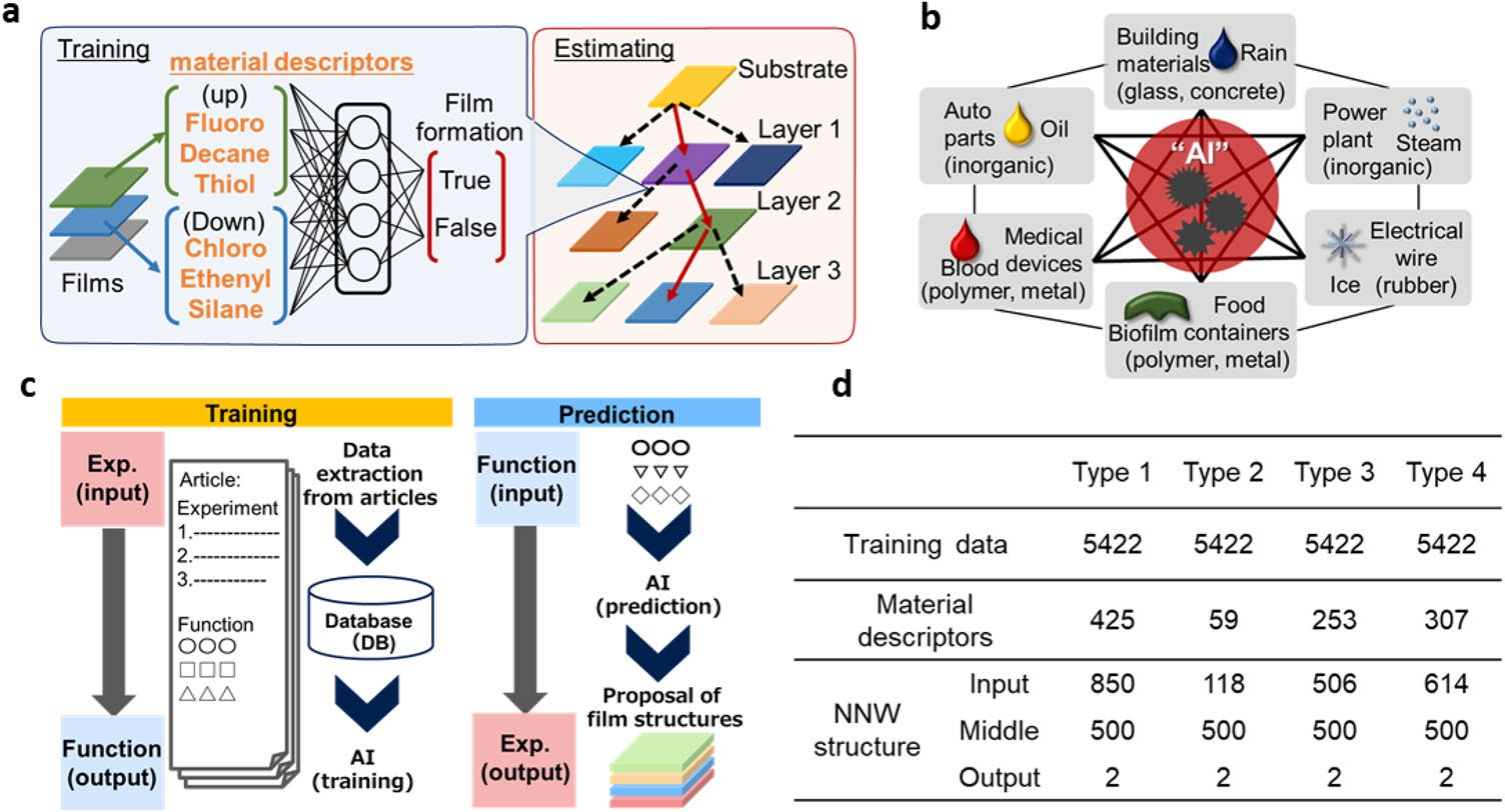
Machine learning method for multilayer functional films. (**a**) Starting from substrate, CNNWs estimate upper layer’s material on down layer, and this procedure is repeated until the end of outermost surface. (**b**) Strongly connect scientific knowledge such as antifouling films with different specialties. (**c**) Whole system for learning and inference. d, Summary of training datasets and NNW structures.

We collected 300 film structures from academic articles (see Methods and Section S3 for reuse permission) and designed multilayer functional films by ML. The entire training and inference process is outlined in Fig. 1. Most of the research articles contained an experimental section and presented new functions, and here we considered the experimental process as input data and functions as output data. These data were extracted by hand and then stored in a database. To clarify the characteristics of each material, we used material descriptors with functional groups (see Fig. S2 and S3 for details). After these relationships had been learned with AI, the relationships between input and output data were used reversely through an inference process. In other words, an experimental procedure for multilayer film was proposed by inputting an arbitrary function. The datasets and NNW structures used in this study are summarized in Fig. 1d.

### Training of multilayer functional film structures

**Figure 2:**
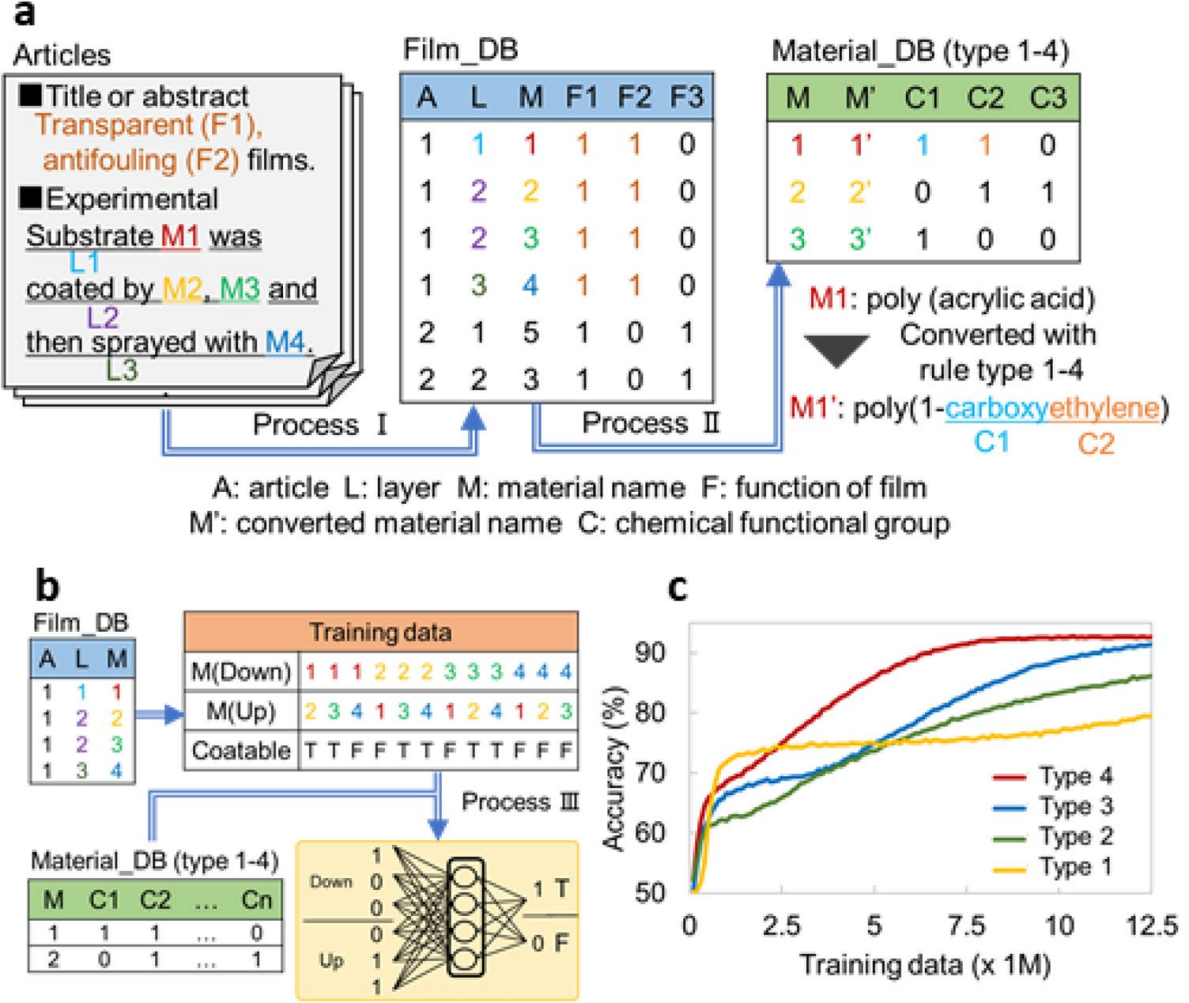
Data collection and learning by different material descriptors. (**a**) Data extraction process for layered structure and functionalities. (**b**) Process for learning film formation property through training data. (**c**) Accuracy of judgment results with four different material descriptors with increasing total training data.

We prepared training data using the following procedures: First, layered structures (= 300) and functionalities (= 26) were stored in the Film_DB by hand (see Fig. 2, Table S1 and S3). Each fabrication step in the scientific articles was separated according to terms related to coating such as ‘dipped’, ‘sprayed’, and ‘deposited’. Then, related materials in each sentence were stored. The second procedure was to convert material names with material descriptors in the Material_DB (see Table S2 and S4). Each substance was unified with IUPAC naming rules and then characterized with material descriptors to add feature values by one-hot encoding (nominal scale), which was done to determine whether part of the material name matched the classification by the descriptor. The last procedure was to generate training data derived from the combination of two materials in the film. For example, a three-layer film (Layer 1, A, B; Layer 2, C, D; Layer 3, E) was separated into A–B, A–C, A–D, B–A, B–C, B–D, C–D, C–E, D–C, D–E as coatable (true) and A–E, B–E, C–A, C–B, D–A, D–B, E–A, E–B, E–C, E–D as uncoatable (false) (see Methods and Fig. S4). Then, the NNW learned the film-forming properties between two randomly selected substances. After classifying 297 structures for training and three untrained structures as test samples for hold-out validation (see section S5), it learned the material pairs in 5,422 data derived from 297 structures. We monitored its accuracy, namely whether the AI output fitted the training data, as total training data increased (Fig. 2c). Here, we compared four types of material descriptors (see Methods for details). Type 1 was the simplest; however, it was difficult to train fine relationships due to its low number of feature values. Others had higher accuracy because they had more feature values. Every material relationship was surveyed through AI (see Fig. S5). Originally, 47.5% of material pairs from 5422 training data had film-forming properties. A higher percentage from the border value meant that AI tended to output false positives; a lower percentage meant it tended to output false negatives. Among the four types of material descriptors, type 3 and 4 showed relatively fast convergence and were considered suitable for learning, whereas others showed turbulence and unstable results.

### Inference of multilayer functional film structures

**Figure 3:**
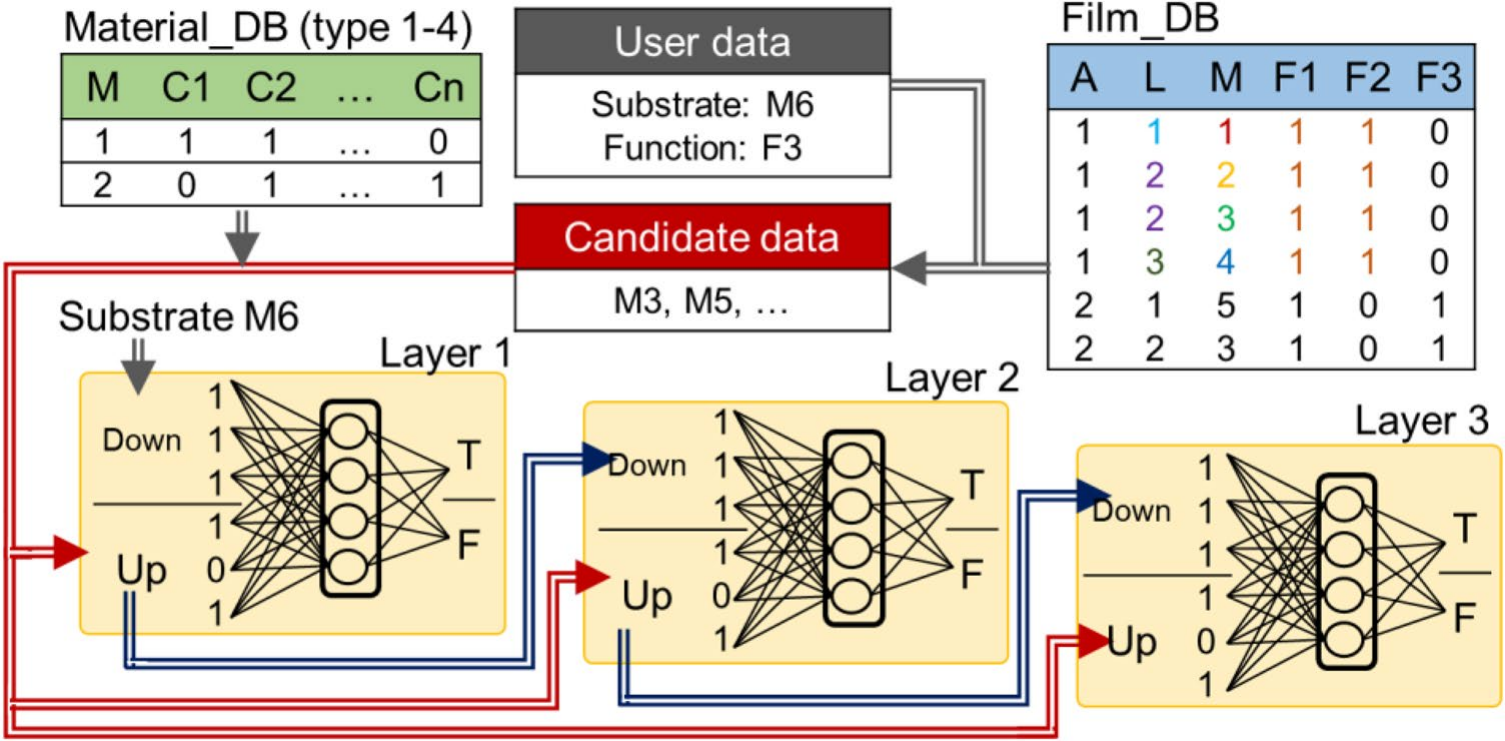
Prediction of multilayer functional films with CNNWs. Connected NNWs plan multilayer film structures from substrate to each layer in order. Through these processes, upper materials (latter process) on lower ones (former process) were gathered as stacked-layer information.

To estimate multilayer functional film by ML, we propose CNNWs, a cascade connection of multiple NNWs (see Fig. 3, S6 and S7). Here, we use the same trained NNW in CNNWs. Prediction procedures were designed as follows: First, ‘User data’ containing the user-defined substrate and functions and ‘Candidate data’ with material names extracted from “Film_DB” fulfilling above functions were prepared. Then, material names converted by a material descriptor with Material_DB were entered into CNNWs. Starting from the substrate, the material of each layer was predicted, and this procedure was repeated until the outermost surface. Finally, CNNWs proposed a layered structure with user-defined functions.

**Figure 4:**
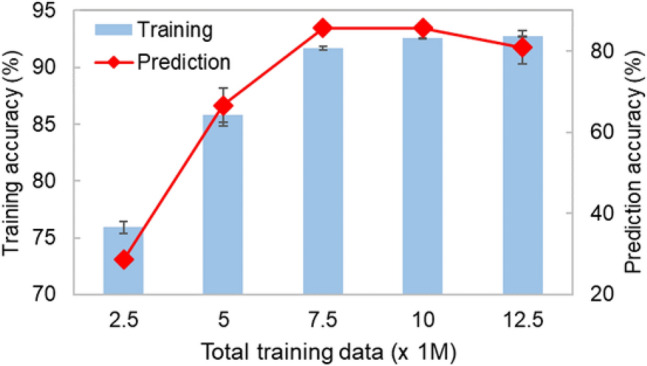
Optimization of training number. Analyzation accuracy for untrained films with type 4. Prediction accuracy was calculated through count of the true number divided by total interface (= 14 in Table S5). When prediction accuracy decreased at 12.5 million, over learning was observed.

To verify the concept of multilayer film design by AI, we first assessed the film stacking property at each interface. The trained NNW judged the film-forming property of each untrained layer (see Table S5). Every prediction result should be true because all test data were from published articles, except for material pairs marked with asterisk (*), which means that they were not in untrained films. However, the prediction for those materials is also expected to be true, because there is a hydrophobic-hydrophobic interaction. Material pairs labeled with two asterisks (**) mean that these were not contained in training data. The type 4 material descriptor, which used the multiple features, showed the highest accuracy for predicting untrained data. Other descriptors gave relatively low accuracy, so type 4 was considered to be suitable for inference. Therefore, ML was performed with this descriptor in subsequent statements. Moreover, there seemed to be an appropriate number of trainings (Table S5), and we summarize the relationship between number of trainings and estimation performance in Fig. 4. From the above results, less training data led to misjudgment, and too much training data also caused deterioration, namely it led to a lack of flexibility due to over-fitting. In particular, type 4 with 7.5 or 10.0 million total training data (over 5000 training data were used for learning many times, allowed duplication) showed the highest estimation performance, including for material pairs labeled with * or **. This is because we considered that related materials were also able to be proposed by using functional groups as material descriptors. For example, material pair No. 109/No. 37 or No. 42 was expected to have a hydrophobic-hydrophobic interaction through “fluoro”. No. 390/No. 224 material pair had Coulomb force between negative charge from “carboxy” and positive charge from “amino”. This kind of pair might be contained in training data. Another pair, No. 124/No. 125, had a similar relation in trained data through “specific polymer - silver” such as No. 124/No. 19 (silver/trifluoroacetate) in Article No. 146.

**Figure 5:**
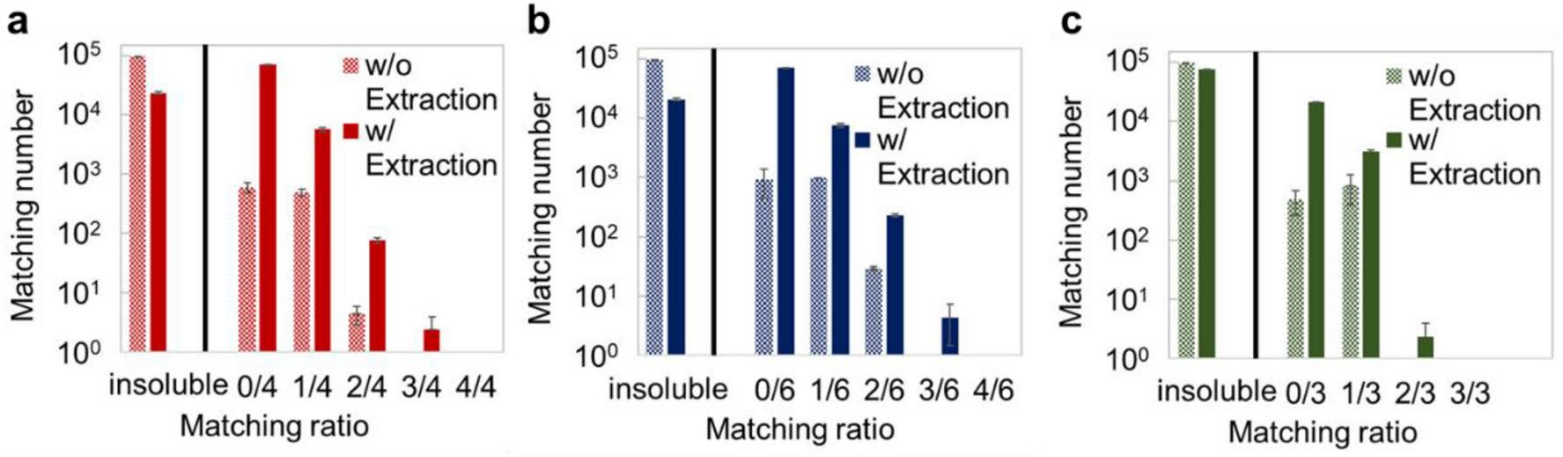
Estimation results with CNNWs. Prediction results for untrained article. **(a**) No. 46, (**b**) No. 180, (**c**) No. 39. Insoluble means AI was unable to propose function-fulfilling structures. Others showed function-fulfilling structures with different matching ratios between AI output and the untrained film’s structure.

We considered that the reason for the highly accurate judgment in type 4 was the utilization of the article’s relationship (see Fig. S8 and S9). Untrained articles as test samples had potential to be predicted by training data through ML. We considered that combining various designs has potential to lead to new designs. Finally, we predicted the whole structures of three untrained films through CNNWs. Figure 5 shows matching ratio between results from AI output and untrained film structures. By using candidate data extracted from training data with limited functionalities defined by the user, enhanced film estimation performance was observed. In the case of type 4, training data’s material (= 425) was reduced to candidate data (= 227). CNNWs proposed for instance, Cellulose—trichlorovinylsilane—perfluorodecanethiol—toluene—perfluoroalkylether in the test of article No. 46. This structure was almost the same with untrained test data. We also demonstrated the design of an original functional film based on this concept (see section S6). This method is a unique estimation approach for multilayer structures, and it is available for various complicated structures with multiple material interfaces or fabrication steps, which data-driven approaches now have difficulty estimating. Thus, this technology offers a new way for designing multilayer structures in materials science.

## Methods

### Data collection from academic articles and labeling

We gathered articles on multilayered films especially with antifouling property, because it was surface modification technology having tendency to collaborate with some functions and widely used in daily-life products (see Fig. 1b and Section S4). By selecting ‘hydrophobic’ or ‘hydrophilic’ as main keywords, we manually collected about 200 papers containing 300 film structures. We surveyed their film fabrication process (the details are shown in Fig. 2a and b) and stored film structures and functions in a database named Film_DB. Substances for coating were converted with IUPAC rules through PubChem (https://pubchem.ncbi.nlm.nih.gov/). Each name was characterized with four types of material descriptors newly defined as follows: using each material names as is (type 1); IUPAC names classified through functional groups with hypernym, a higher level concept, such as alkyl group (type 2) and with hyponym, a lower level concept, such as methyl group (type 3); or with both higher and lower level concepts (type 4) as shown in Fig. S2 and S3. Materials with characterized information were stored in a database named Material_DB. Finally, films were classified for 297 training and three untrained structures to assess the estimation property by the hold-out validation method.

### Training material stacking property between two substances by NNW

From 297 training structures, we made 5422 training data related to the stacking properties of material pairs (see Fig. S4). Briefly, materials of adjacent layers limited to those in ascending order and materials in the same layer were defined as True. On the other hand, materials with descending order or materials of distant layer were set to False, because they had no track record of film stacking. If the same pair had different film-forming properties, priority was assigned to “True”. Through this process, 5,422 material pairs were formed. Their relationships (whether upper layer’s material can be coated on a lower layer’s material or not) were learned by the NNW on ML software Tensorflow (https://www.tensorflow.org/). The NNW was organized as follows: The input layer was double the amount of all material descriptors. The middle layer and output layer were fixed to 500 and 2, respectively (see Fig. 1d). From training data, 100 randomly selected data were used for learning and then the accuracy was checked by another one hundred data. By repeating this process, the layer-forming property was learned.

### Estimation of multilayer functional film structures by CNNWs

We used untrained films for hold-out validation. After the above training, we extracted candidate data from training data that had potential to achieve an untrained film’s functions. Starting from a substrate, the upper layer’s material was estimated by inputting data composed with two materials (upper and lower layer’s material) into the NNW, and this procedure was repeated until the end of outermost surface. Finally, the film was judged as to whether the materials met all functions of untrained film.

## Conclusion

We proposed designing multilayer functional films by ML of chemical-coating articles. The technology features CNNWs that connect multiple NNWs. For data preparation, 300 film structures were collected from 200 papers, and constituent substances were converted with IUPAC naming rules and then characterized with four types of material descriptors. By comparing descriptors, classification through functional groups (type 4) with training performed 7.5 or 10.0 million times was found to be suitable for learning (Fig. 2c) and inference of the relationship between the two materials (Table S5 and Fig. 4). Then, we showed the prediction results of untrained film structures by CNNWs of type 4. Moreover, enhanced film estimation performance was observed by squeezing candidate data (Fig. 5). This technology represents a new way to design complex structures related to daily-life products by connecting scientific literature.

## Supplementary Information

Supplementary Information.
