# Supplementary material for: Designing a multilayer film via machine learning of scientific literature
Source: Sci Rep. 2022 Jan 18;12:930. doi: 10.1038/s41598-022-05010-7 (PMC8766440; doi:10.1038/s41598-022-05010-7)
Supplement: Supplementary file 1 — Supplementary Information. [file 41598_2022_5010_MOESM1_ESM.docx]

Supplementary Information

Designing a multilayer film via machine learning of scientific literature

Kenta Fukada* and Michiko Seyama

NTT Device Technology Labs, NTT Corporation

3-1 Morinosato, Wakamiya, Atsugi, Kanagawa, 243-0198, Japan

**S1 Image of concept for designing a multilayer functional film**


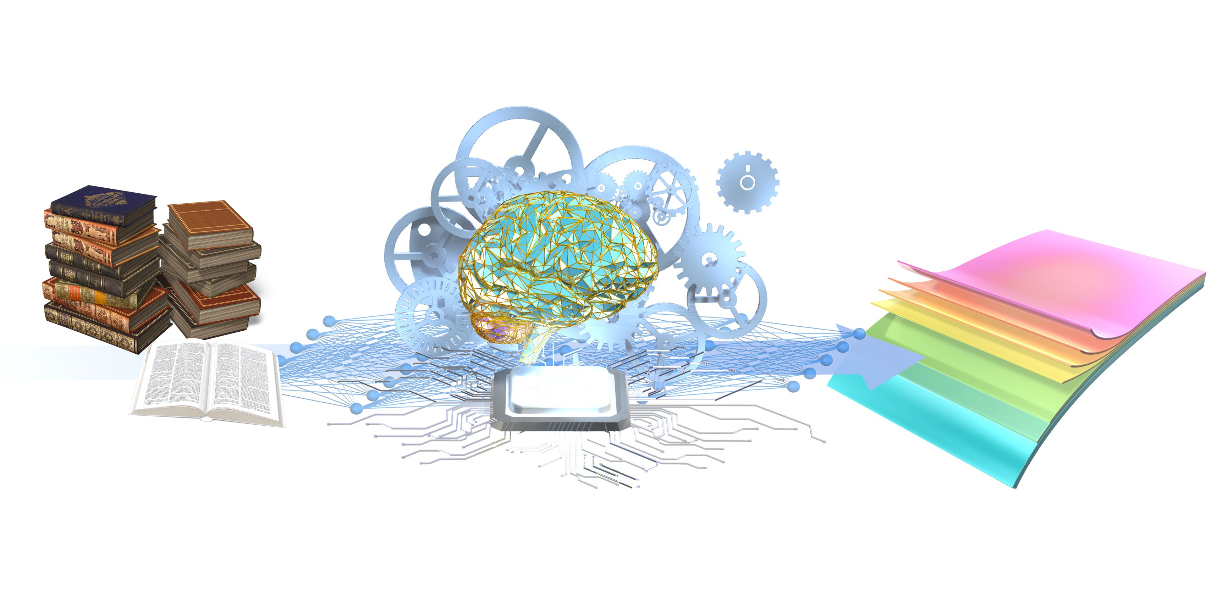


**Fig. S1: Design process via machine learning of scientific literature.** Through machine learning of experimental procedures extracted from chemical-coating articles, a multilayer film can be designed through an inference process.

**S2 The reason for using neural network**

We used NNWs instead of other technologies, such as support vector machines and random forest, for their ease of implementation and high extensibility.

First, we can execute learning and inference with extremely simple code and easily change the number of intermediate layers by using Tensorflow, etc. Then, we can focus on other parts such as input/output data-shaping processes. In this research, we proposed a multistage NNW platform, but in actual work, it takes more time to prepare the data-shaping process than it does the main part.

Second, we classified film formation into two cases, namely whether it was possible or not, but it was considered that we would be able to further classify film formation properties (N class) in the future. In actual film growth, the amount of adsorption or film thickness changes depending on the compatibility of, for instance, surface functional groups, and there are cases where the film is hardly attached or excessively attached. An NNW can deal with such cases (for example, classification by film thickness) by simply changing the number of classifications of the output layer.

**S3 Reuse permission**

The references at the end of this Supplementary Information show each article we used for training and inference. All papers were purchased, and permission for reuse in a journal was obtained thorough the Copyright Clearance Center (RightsLink^®^). In this study we used titles, abstracts, or excerpts within 400 words to make training and　prediction datasets.

**S4 Data preparation and training process**

We collected articles mainly related to antifouling films. This theme was selected because such films consist of multiple substances to form layered structures with multiple functionalities. Traditionally, they have been designed by conducting a large number of experiments, and it has been difficult for researchers or technologists to utilize data-driven approaches. Research papers covering antifouling films have been published in various journals, and the films have been applied in industrial domains such as building materials, medical devices, and auto parts [(Fig. 1(b)]. We considered that these had some potential as a data source. If scientific knowledge from various specialties can be leveraged by combining the multi-functionality layer information in AI, it will provide a critical designing tool. Thus, we proposed a machine learning method for planning multilayer functional films with scientific literature.

For the training process, the details of data extraction and storage are shown in Fig. S2. Chemical functional groups are summarized in Table S1 and S2. We focus on functional groups of materials because they tend to be considered as the reasons for selecting materials for stacking multilayer membranes. Thus, we used material descriptors in this study. For instance, ‘cellulose” was transformed to (6S)-2-(hydroxymethyl)-6-[(3S)-4,5,6-trihydroxy-2-(hydroxymethyl)oxan-3-yl]oxyoxane-3,4,5-triol by the IUPAC naming rule, and this was used as is (type 1 material descriptor). Type 1 was further classified through functional groups with a higher level concept and featured as Cyclic compound, Substituent, Alkyl, Ether, Oxygen content, and Heterocyclic group (type 2). Type 1 was also classified through functional groups with a lower level concept as hydroxy, methyl, oxanyl, oxy, oxane, and ol (type 3), By mixing type 2 and 3, cellulose was featured with Cyclic compound, Substituent, Alkyl, Ether, Oxygen content, Heterocyclic group, hydroxy, methyl, oxanyl, oxy, oxane, and ol (type 4). Sample data are shown in Table S3 and S4. The training data preparation process is explained by the flowchart in Fig. S4.


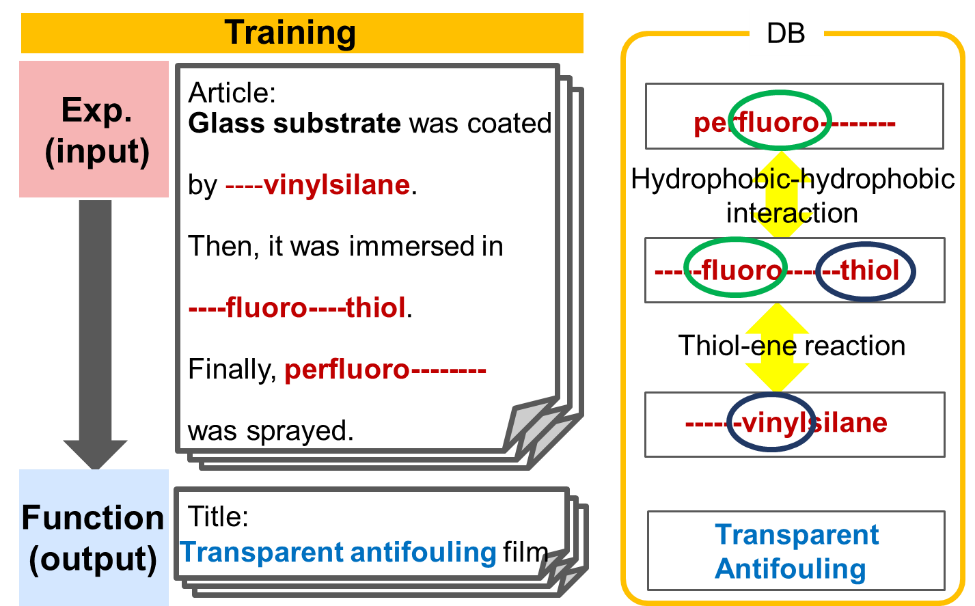


**Fig. S2: Data extraction and database storage process.** The name of the stacked material, the order of lamination process and the function of the film were extracted from the manufacturing processes in papers or paper titles and then these data were stored in the database. In most of the coating processes, there were reactions mediated by functional groups such as thiol-ene reaction and hydrophobic-hydrophobic interaction. Therefore, we used functional groups in the material’s name by referring to IUPAC naming rules, which were able to express them.


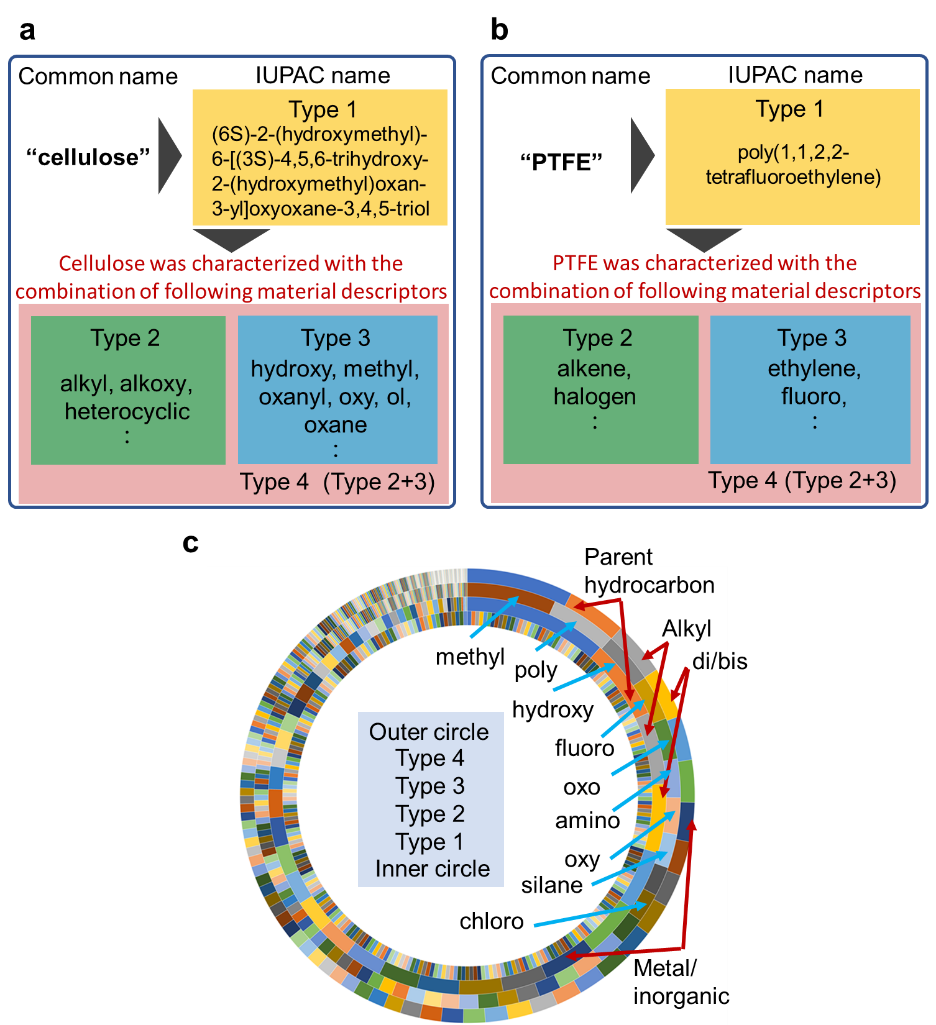


**Fig. S3: Material descriptors. a,** Material names were classified by one-hot encoding (nominal scale) with International Union of Pure and Applied Chemistry (IUPAC) rules. These descriptors were based on functional groups, but amine and carboxyl groups can, for instance, be included in anionic and cationic materials; thus, we added surface charge information. Polymer information (poly) and the number of name splits (1 – 12), the number of regions in the material name that match the material descriptor, were also used. Four types material descriptors were defined as follows: Using each material name converted with IUPAC naming rules as is (type 1); or IUPAC names classified through functional groups with a higher level concept (type 2), with lower level concept (type 3); or with higher and lower level concepts (type 4). **b,** The case for PTFE. **c,** Arc length shows the material descriptor ratio for 425 materials from 297 film structures. Total numbers of material descriptors in the Material_DB were 3,973, 1,436, 2,583, and 425 in type 4, 3, 2, and 1, respectively. For example, ‘Methyl’ appeared 97 times in the database with type 3.

**Table S1: Functions in Film_DB.**


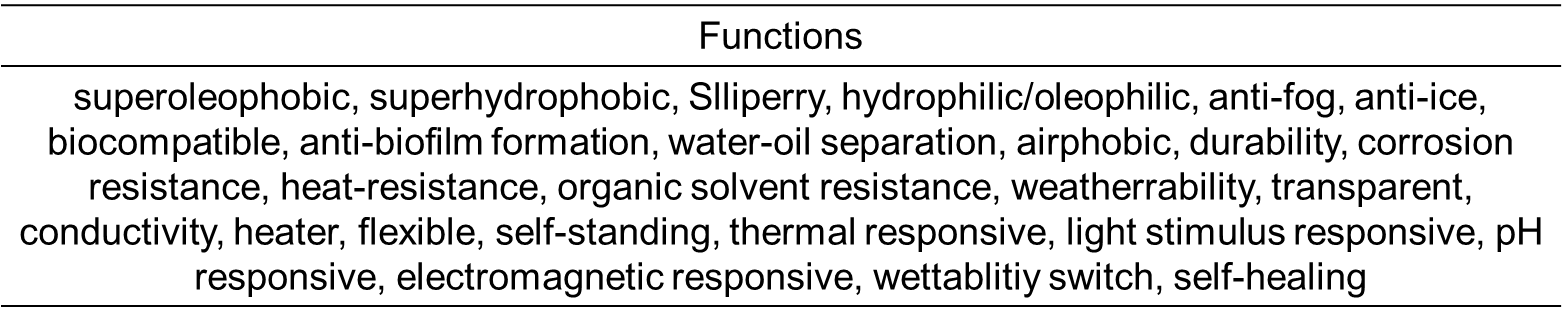


**Table S2: Material descriptors in Material_DB.**


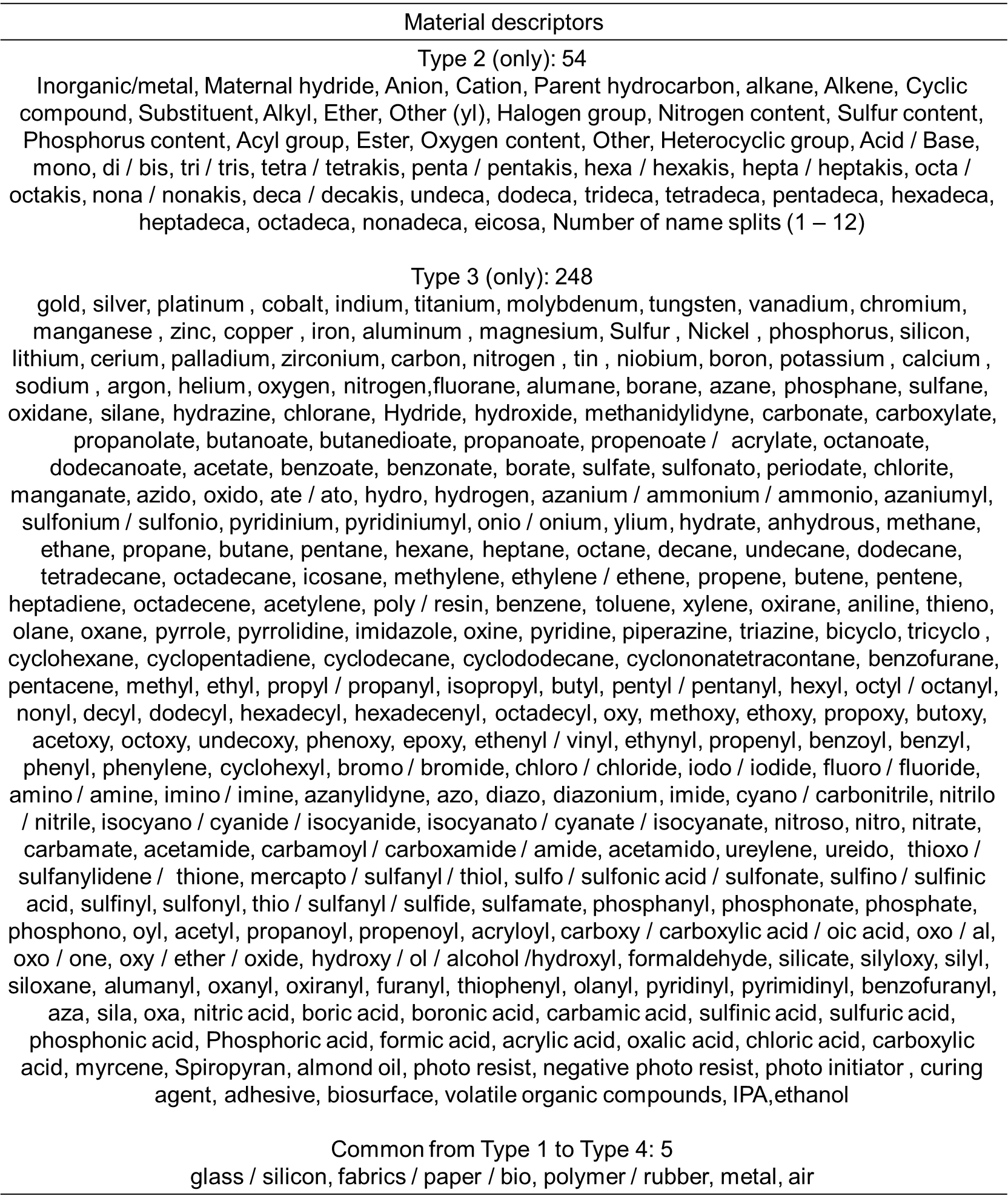


Each type has five common material descriptors [substrates classified into 4 categories (glass / silicon type, fabrics / paper / biomaterial type, polymer / rubber type and metal type), and air (representing above the outermost surface)]. There are 54 material descriptors for type 2 only, so the total is 59. Only type 3 has 248, so the total is 253. Type 4 is a material descriptor of 307 from type 2 and type 3 eliminating duplication (= 59 + 253 – 5).

**Table S3: Sample of Film_DB.**


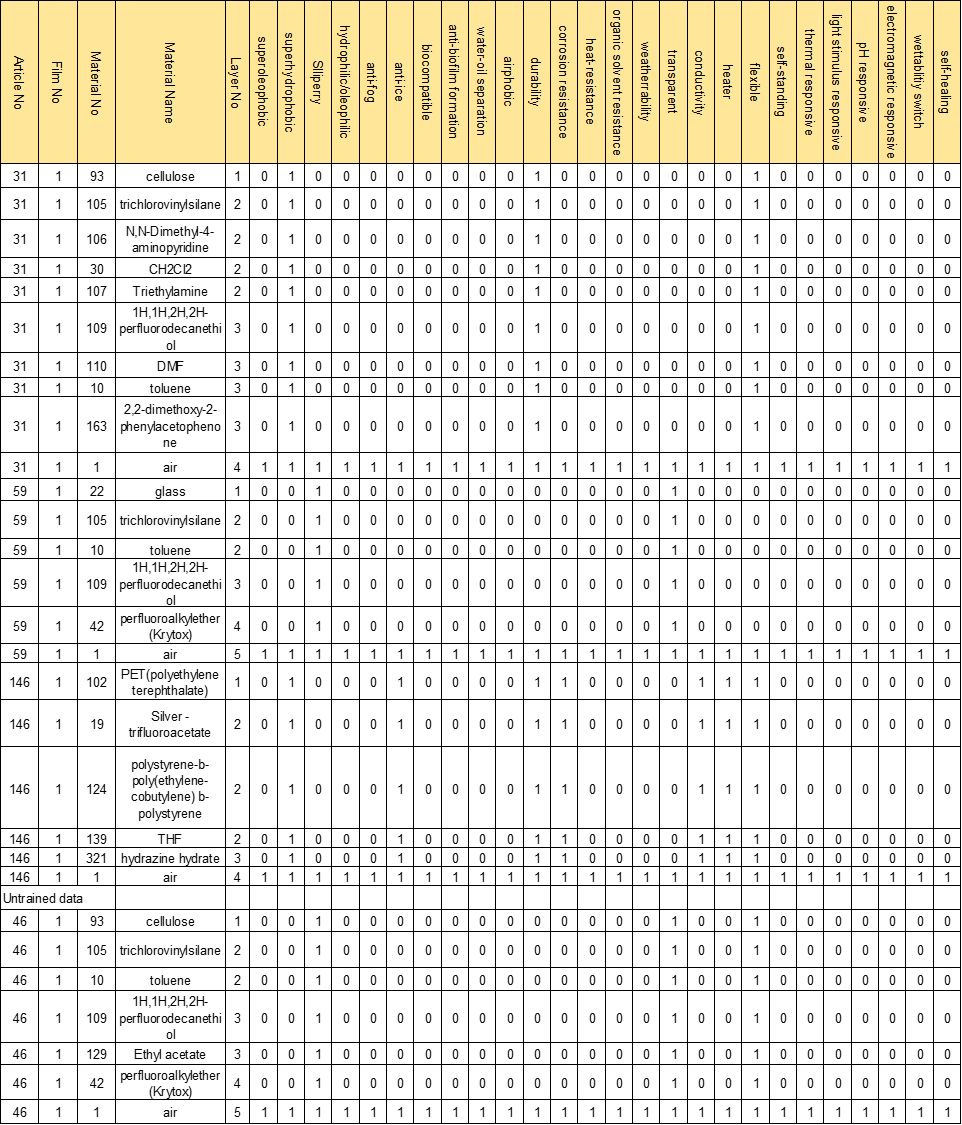


31: Reprinted with permission from Wang, M. et al. Fast Strategy to Functional Paper Surfaces. *ACS Appl. Mater. Interfaces.* **11**, 14445–14456. Copyright 2019 American Chemical Society. 59: Reprinted with permission from Li, J. et al. Reactive superhydrophobic surface and its photoinduced disulfide-ene and thiol-ene (Bio)functionalization. *Nano Lett.* **15**, 675–681. Copyright 2015 American Chemical Society. 146: Reprinted with permission from Wu, L. et al. Durable and Multifunctional Superhydrophobic Coatings with Excellent Joule Heating and Electromagnetic Interference Shielding Performance for Flexible Sensing Electronics. *ACS Appl. Mater. Interfaces.* **11**, 34338–34347. Copyright 2019 American Chemical Society. 46: Reprinted with permission from Guo, J. et al. Superhydrophobic and Slippery Lubricant-Infused Flexible Transparent Nanocellulose Films by Photoinduced Thiol-Ene Functionalization. *ACS Appl. Mater. Interfaces*. **8**, 34115–34122. Copyright 2016 American Chemical Society.

**Table S4: Sample of Material_DB.**


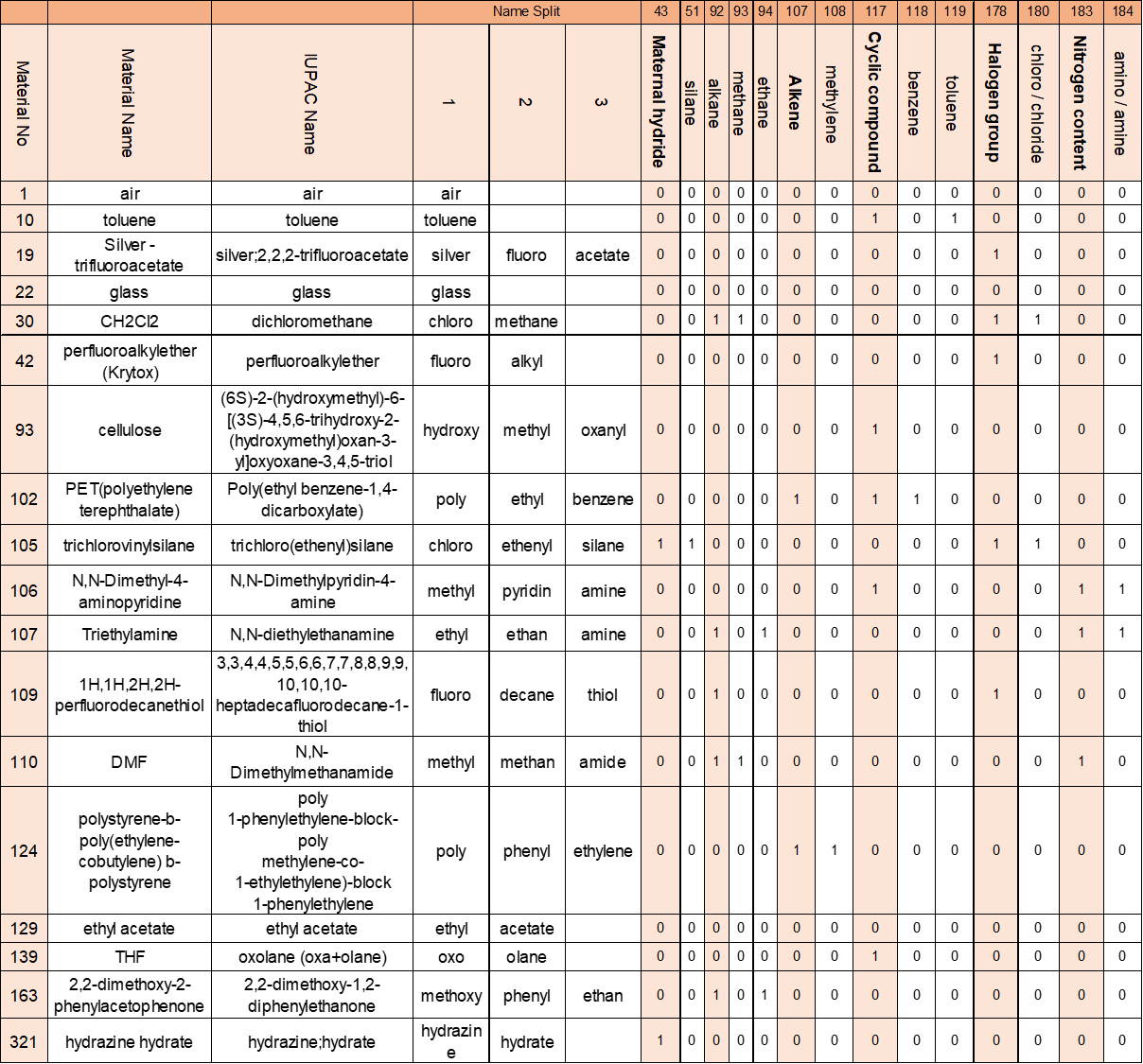


If a part of the material name matches the classification of the descriptor, it is set as “1”; if it does not match, it is set as “0” and converted into learning data. This number is a nominal scale; the numbers themselves and the order have no meaning.


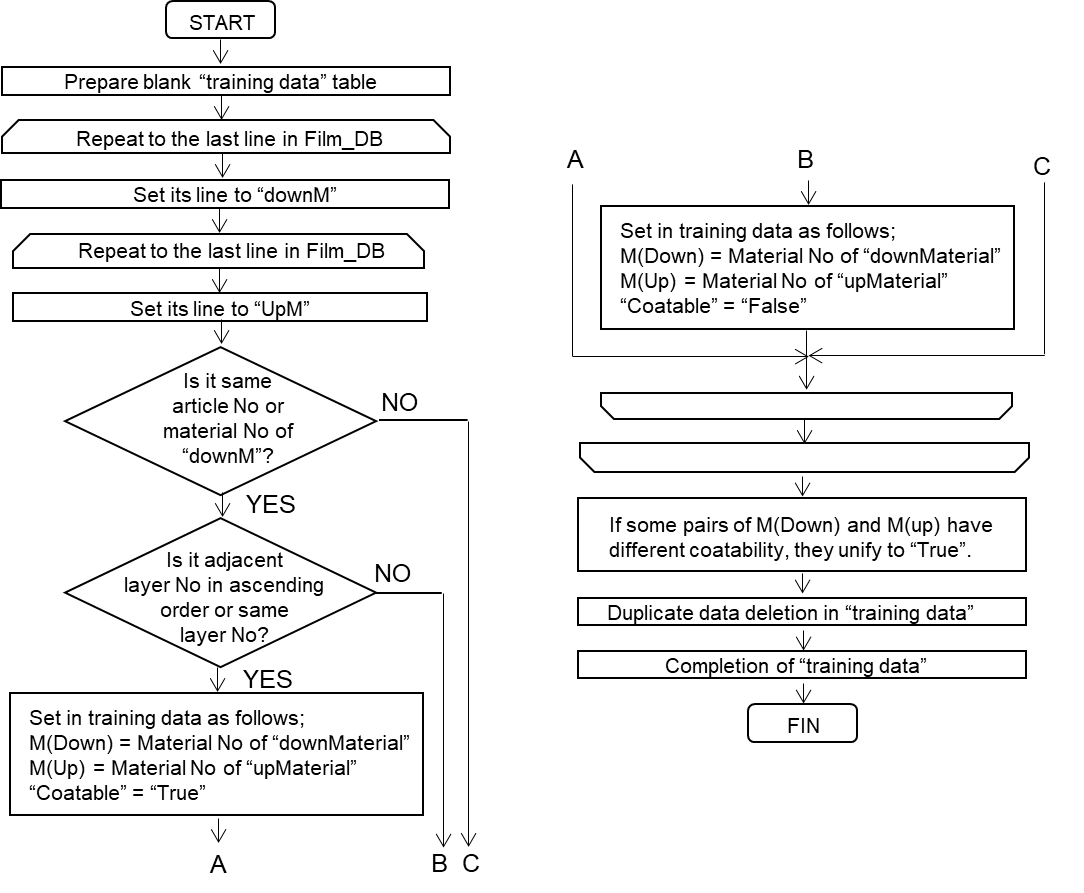


**Fig. S4: Training data preparation.** This is a process for converting the multilayer data extracted from the articles into data for each of the two layers. Our interpretation is that the relationship between the materials of the upper layer and the lower layer can form a film, but the opposite is not always possible. For example, hydrophobic oil can be dropped onto a hydrophobic film to form an oil film, but it is rare to form a hydrophobic film on the oil. Therefore, such reverse film formation is set to false. However, if the learning data include instances where such a process was tried in past research, it can be overwritten as true. As a concrete process, from “Film_DB”, an adjacent layer’s materials　limited in ascending order, and same layer’s materials were defined as true. On the other hand, materials with descending order or distant layer’s materials were set to false. If the same pair had a different film-forming property, priority was assigned to “true”. Through these processes, over 5,000 material pair patterns were formed. Then, the NNW was trained with such pairs of materials as input data and the true/false results as output data.


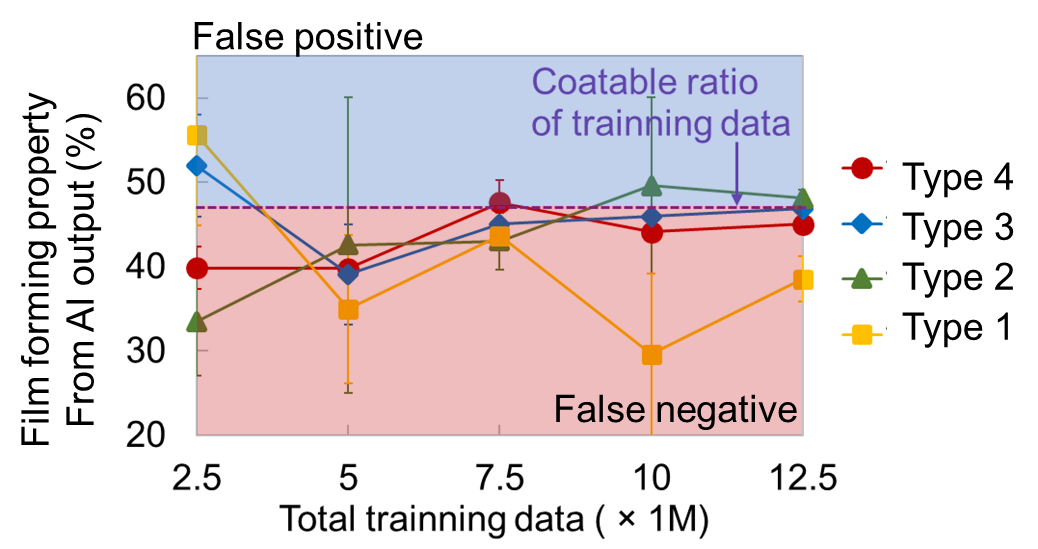


**Fig. S5 Results of trainings.** All training data were judged with NNW. Originally, 47.5% of training data had film-forming property (materials in adjacent layer or same layer) as shown by the purple dashed line. Higher than this line means that AI tended to output false positives; and lower means false negatives. Type 1 and 2 showed turbulence and unstable results.

**S5 Inference process**

At present, there is no critical data collection tool for our concept, so we need to gather training data by hand. Because of the low amount of training data, it was difficult to conduct cross validation. However, to maintain prediction accuracy at some level, we used hold-out validation, separating training and test data.

Regarding overfitting, we have confirmed that the results in Table S5 were limited to the learning data when the material name itself was learned (materials descriptor type 1) or the number of learnings increased too much. Therefore, we divided the material name into functional group information and estimated the appropriate number of learning times. Of course, it is not possible to handle all cases, and improvement is necessary, so we will continue verification under various conditions in combination with data collection tools using natural language processing to increase input data.

**Table S5. Estimating results from NNW for each layer**


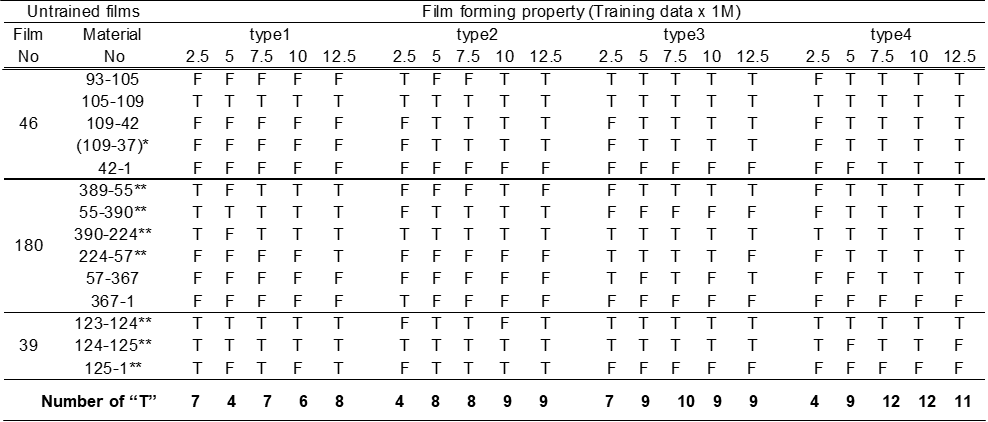


Film forming property (Ture or False) at each interface were shown. Materials with * and ** were not in test and training data respectively. 93: cellulose, 105: trichlorovinylsilane, 109: perfluorodecanethiol, 42: perfluoroalkylether, 37: Fluorinert, 1: Air, 389: poly ethylene terephthalate glycol modified, 55: poly ethylene imine, 390: Carboxymethylcellulose, 224: chitosan, 57: 1-ethyl-3-(3dimethylaminopropyl) carbodiimide hydrochloride, 367: N-Hydroxysuccinimide, 123: Natural rubber, 124: poly styrene -b-poly ethylene-cobutylene -b-polystyrene, 125: 1-octadecanethiol modified Ag nanoparticles. 46: Reprinted with permission from Guo, J. et al. *ACS Appl. Mater. Interfaces*. **8**, 34115–34122. Copyright 2016 American Chemical Society. 180: Reprinted with permission from Park, S. et al. *ACS Appl. Mater. Interfaces*. **10**, 17714–17721. Copyright 2018 American Chemical Society. 39: Reprinted with permission from Su, X., Li, H., Lai, X., Chen, Z. & Zeng, X. *ACS Appl. Mater. Interfaces.* **10**, 10587–10597. Copyright 2018 American Chemical Society.


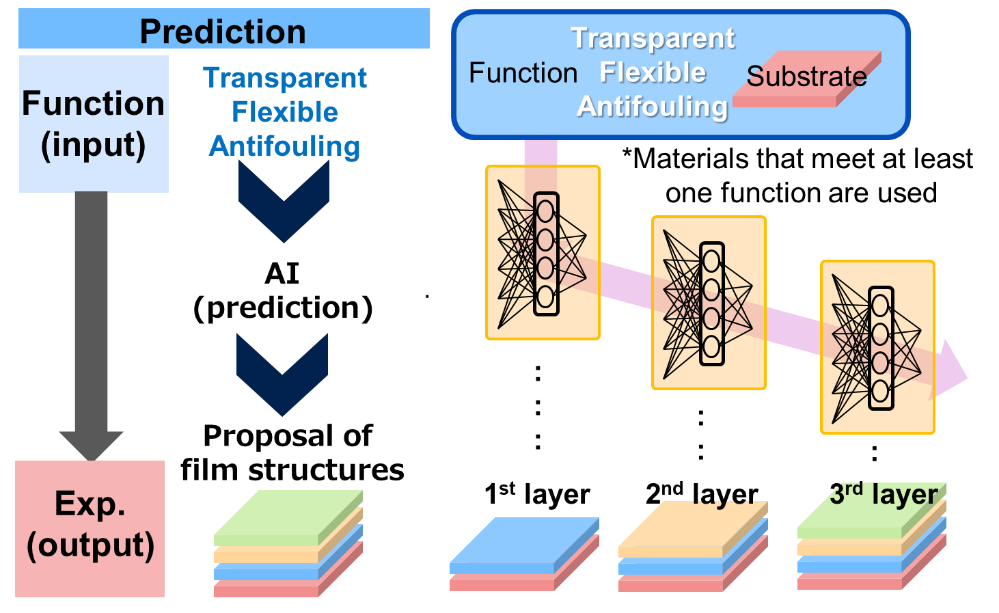


**Fig. S6: Prediction process for multilayer film.** Schematic diagram of inference process for estimating film structure. First, the user enters information into the system about the substrate and the desired features. Data processing is performed as follows. After candidate materials that may satisfy the functions (materials used in the membranes that meet functions) are prepared, AI enters information about the substrate and candidate materials into the NNW, and if they are stackable, enters the next material. By repeating this procedure, AI proposes a multilayer structure and checks whether all the desired functions are satisfied by combining the possible functions of the materials. AI’s job is to propose multilayered structure with the above flow. Then, a person makes a judgment as to whether or not the film is feasible.


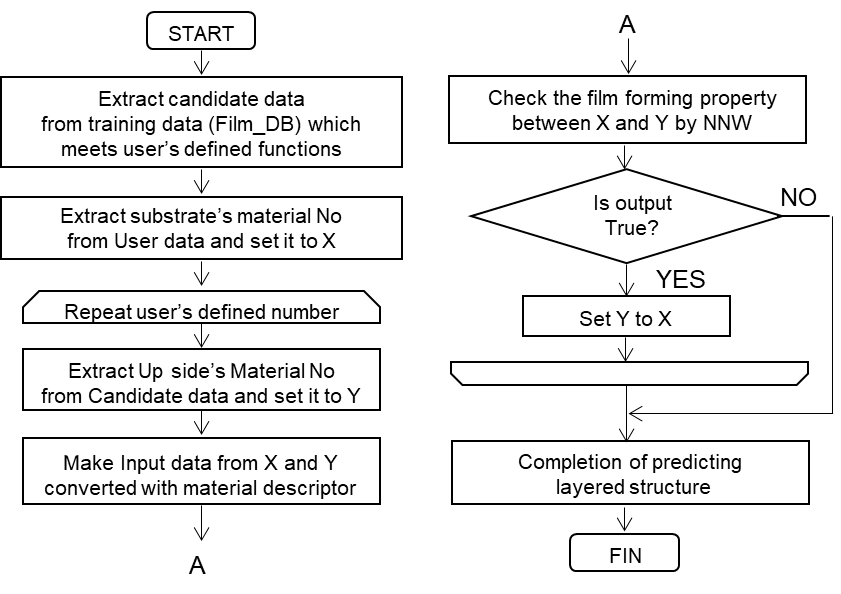


**Fig. S7: Prediction method by CNNWs.** Candidate data is extracted from training data which had potential to achieve desired functions. Input-data composed of two materials from candidate data are analyzed by CNNWs, which connect multiple NNW, and their film formation property is judged repeatedly. Starting from user-defined substrate, upper layer’s material is estimated, and this procedure is repeated until the outermost surface. Finally, the film is judged as to whether the materials it contains meet user-defined functions.


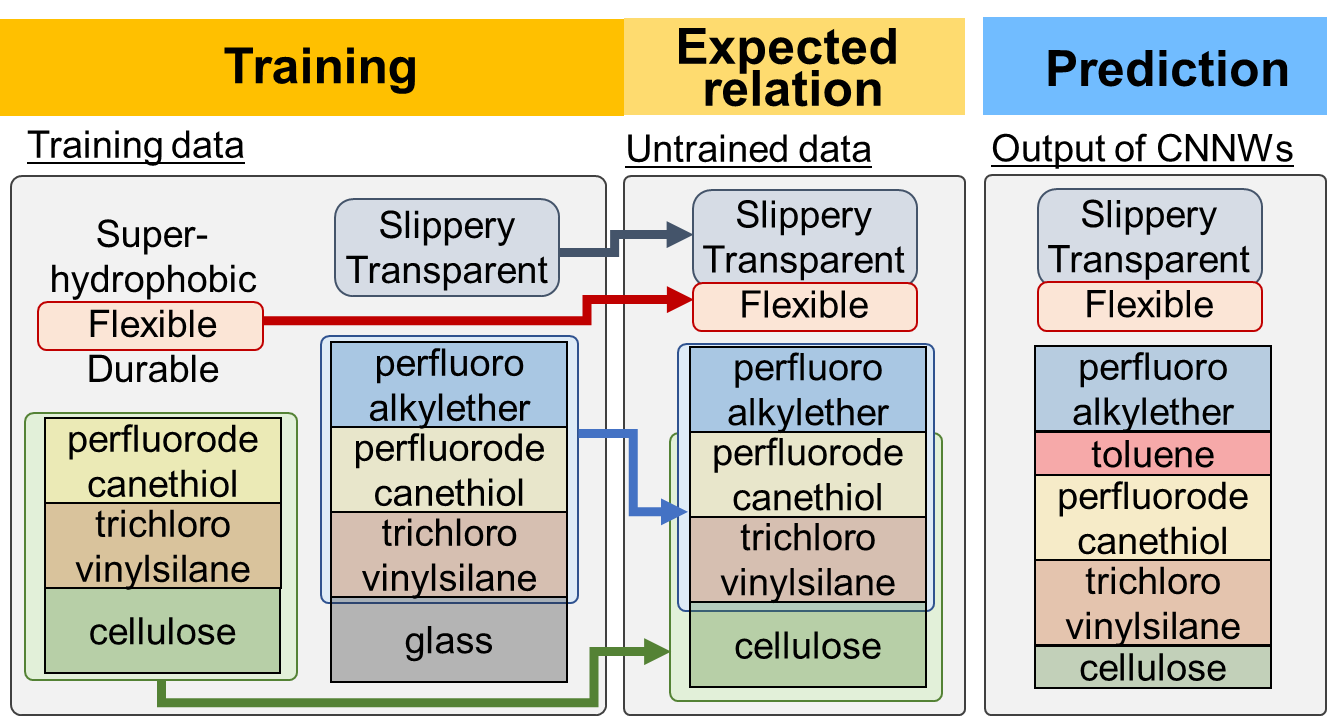


**Fig. S8: Prediction concept through scientific articles.** Schematic diagram for predicting one of three untrained data is shown. Each function and layered structure from training data is expected to contribute to designing untrained data.


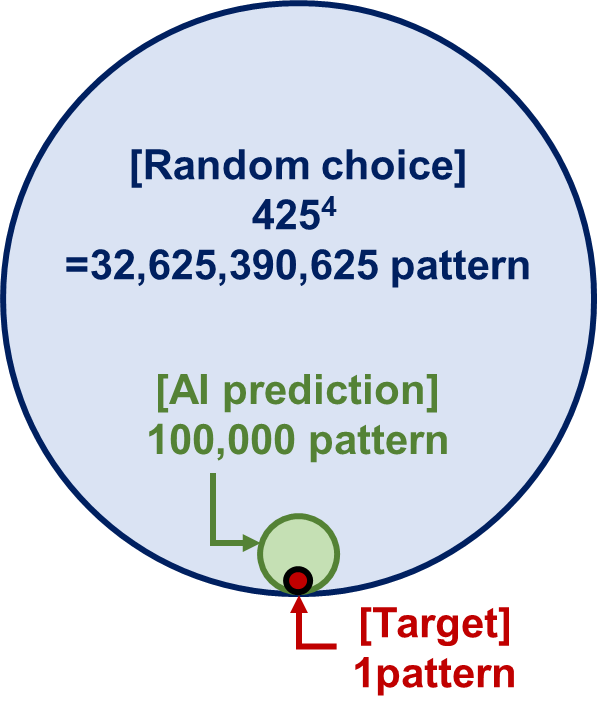


**Fig. S9: Relatively high search performance by AI.** In the case of Film No. 46 in Table S5, there were up to 425^4^ patterns when four layers were randomly examined using 425 materials in the Material_DB. Compared with random choices, we obtained results close to the target structure in 100,000 AI attempts.

**S6 Demonstration**

**
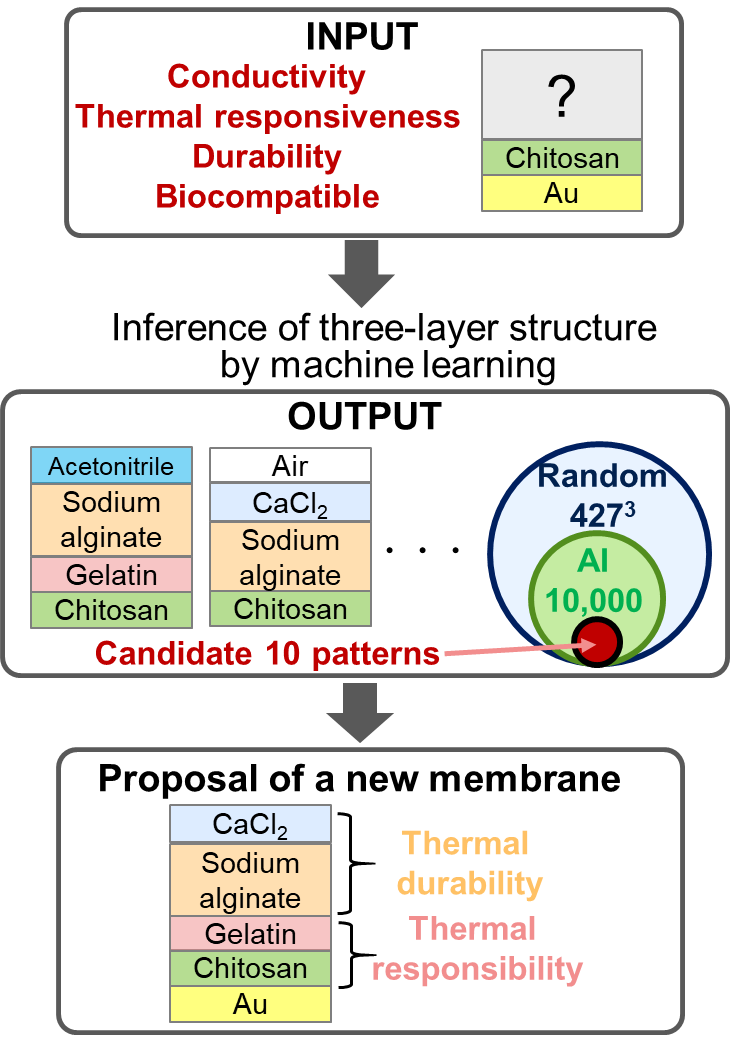
**

**Fig. S10: Demonstration of a film structure design using AI (CNNWs).**

INPUT: A method of proposing what materials can be laminated on chitosan and gold when designing a film with conductivity, thermally responsiveness, durability, and biocompatibility for producing electrodes that naturally disappear in a specific environment. In the demonstration process, we added gelatin and CaCl_2_ data to the database and performed an estimation of the three-layer structures.

OUTPUT: CNNWs output some useful candidates, which were related to combinations of thermal responsive materials or gelation additives, etc. Compared with random choices (427^3^ patterns for three layers), we obtained ten useful results in 10,000 AI attempts.

Proposal of a new membrane: Using the AI output as a reference, we inferred the structure and function of the new membrane. The candidate materials were food and food additives, and by combining materials with different thermal responses, complex functions were expected to be achieved.

**
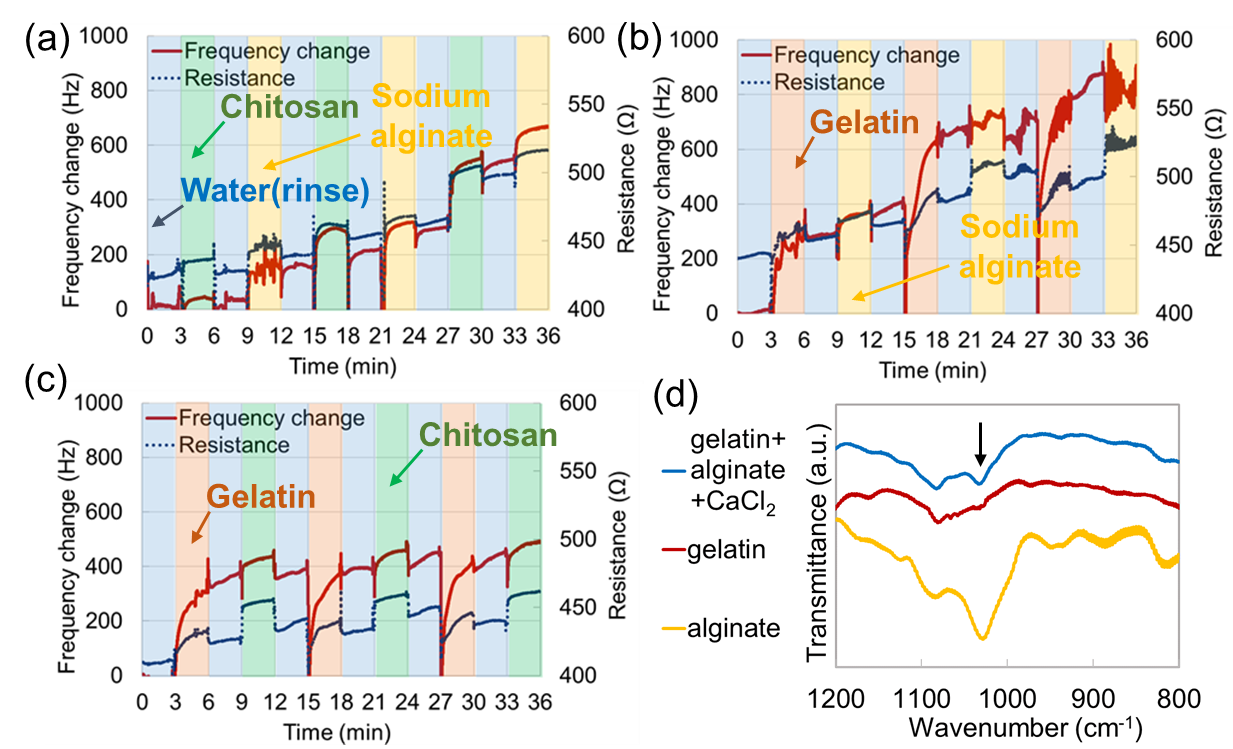
**

**Fig. S11: Confirmation of film formation at each layer.** (a) Quartz crystal microbalance (QCM) measurement of positively charged chitosan aqueous solution (0.1wt%, acetic acid) and negatively charged sodium alginate aqueous solution (0.1wt%). By alternately immersing QCM in cation and anion solution, the amount of adhesion (frequency change) and the viscoelasticity (resistance) of the film gradually increased, indicating that lamination of these materials was possible. (b) A pair of gelatin aqueous solution (0.1 wt%) and sodium alginate was also possible. (c) In the case of gelatin and chitosan, lamination was not possible because they were positively charged by the amine group and could not be stacked due to repulsion. Based on these experimental results and AI outputs, gelatin and chitosan were not used for lamination, but mixed for gelation using gelatin as the main ingredient in this study. (d) The gelatin film could also be laminated by immersion in sodium alginate and calcium chloride aqueous solutions, from the characteristic peak in Fourier transform infrared spectroscopy (FT-IR).


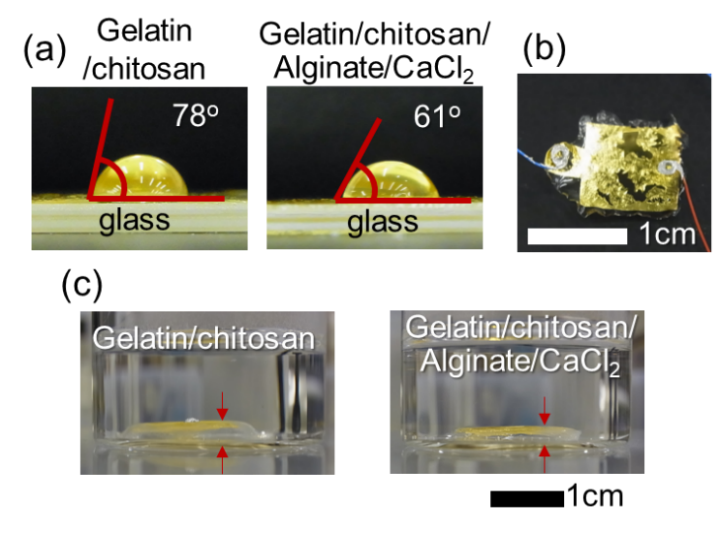


**Fig. S12: Characteristics of multilayer films in demonstration.**

(a) From the change in contact angles, it was confirmed that the surface was modified by stacking these materials. (b) Gel electrodes disintegrated in hot water and showed thermal responsiveness. (c) It was considered that gel swelling was suppressed with sodium alginate/CaCl_2_ in hot water (40^o^ C).

**Limitations and frontiers**

Inference ability was tested with film structures in untrained articles at present; however, it was difficult to confirm the completely new proposed structures. Various film structures suggested by CNNWs require further chemical or physical discussion for practical design. The machine learning process also needs further refinement for material stacking by using annealing temperature, solvent selection, and the material distribution in the vertical direction in the films, etc. For example, material requiring a high temperature in a coating process was difficult to apply to paper substrate. In addition to functional groups, chemical or physical information such as molecular weight or structures should be contained in database. For instance, cellulose’s characteristics differ by molecular weight, but the differences were difficult to distinguish in this paper.

**Outlook**

Although MI holds great promise, there are still obstacles in targeting complex phenomena or chemical reactions. Generally, complex structures are needed in our life because many products are not composed of only a single material but instead require combinations of materials. For the above reasons, there is a need for new data-driven technology for complex structure design. Our CNNWs will contribute to the development of a tool for such complicated structure design in MI.

**References**

1. Han, K., Park, T. Y., Yong, K. & Cha, H. J. Combinational Biomimicking of Lotus Leaf, Mussel, and Sandcastle Worm for Robust Superhydrophobic Surfaces with Biomedical Multifunctionality: Antithrombotic, Antibiofouling, and Tissue Closure Capabilities. *ACS Appl. Mater. Interfaces*. **11**, 9777–9785 (2019).
2. Kang, C. W. et al. Nanoporous Organic Network Coating of Nanostructured Polymer Films with Enhanced Adsorption Performance toward Particulate Matter. *ACS Appl. Mater. Interfaces*. **11**, 1748–1753 (2019).
3. Yu, Y. et al. Highly Stable Amphiphilic Organogel with Exceptional Anti-icing Performance. *ACS Appl. Mater. Interfaces*. **11**, 12838–12845 (2019).
4. Luo, J. et al. Mechanically Durable, Highly Conductive, and Anticorrosive Composite Fabrics with Excellent Self-Cleaning Performance for High-Efficiency Electromagnetic Interference Shielding. *ACS Appl. Mater. Interfaces*. **11**, 10883–10894 (2019).
5. Chen, C. et al. Mimicking from rose petal to lotus leaf: Biomimetic multiscale hierarchical particles with tunable water adhesion. *ACS Appl. Mater. Interfaces*. **11**, 7431–7440 (2019).
6. Guo, H. et al. A Robust Cotton Textile-Based Material for High-Flux Oil-Water Separation. *ACS Appl. Mater. Interfaces*. **11**, 13704–13713 (2019).
7. Yong, J., Singh, S. C., Zhan, Z., Chen, F. & Guo, C. Substrate-Independent, Fast, and Reversible Switching between Underwater Superaerophobicity and Aerophilicity on the Femtosecond Laser-Induced Superhydrophobic Surfaces for Selectively Repelling or Capturing Bubbles in Water. *ACS Appl. Mater. Interfaces*. **11**, 8667–8675 (2019).
8. Xiao, X. et al. Bioinspired slippery cone for controllable manipulation of gas bubbles in low-surface-tension environment. *ACS Nano*. **13**, 4083–4090 (2019).
9. Yan, X. et al. Atmosphere-mediated superhydrophobicity of rationally designed micro/nanostructured surfaces. *ACS Nano*. **13**, 4160–4173 (2019).
10. Luong, D. X. et al. Laser-Induced Graphene Composites as Multifunctional Surfaces. *ACS Nano*. **13**, 2579–2586 (2019).
11. Kim, P. et al. Liquid-infused nanostructured surfaces with extreme anti-ice and anti-frost performance. *ACS Nano*. **6**, 6569–6577 (2012).
12. Agapov, R. L. et al. Asymmetric wettability of nanostructures directs Leidenfrost droplets. *ACS Nano*. **8**, 860–867 (2014).
13. Guix, M. et al. Superhydrophobic alkanethiol-coated microsubmarines for effective removal of oil. *ACS Nano*. **6**, 4445–4451 (2012).
14. Kim, Y. H., Lee, Y. M., Lee, J. Y., Ko, M. J. & Yoo, P. J. Hierarchical nanoflake surface driven by spontaneous wrinkling of polyelectrolyte/metal complexed films. *ACS Nano*. **6**, 1082–1093 (2012).
15. Xu, Q. F., Wang, J. N. & Sanderson, K. D. Organic-inorganic composite nanocoatings with superhydrophobicity, good transparency, and thermal stability. *ACS Nano*. **4**, 2201–2209 (2010).
16. Balogh, D., Tel-Vered, R., Riskin, M., Orbach, R. & Willner, I. Electrified Au nanoparticle sponges with controlled hydrophilic/hydrophobic properties. *ACS Nano*. **5**, 299–306 (2011).
17. Yuan, L. et al. Self-cleaning flexible infrared nanosensor based on carbon nanoparticles. *ACS Nano*. **5**, 4007–4013 (2011).
18. Pei, C. et al. An Integrated Janus Mesh: Underwater Bubble Antibuoyancy Unidirectional Penetration. *ACS Nano*. **12**, 5489–5494 (2018).
19. Yoo, D. et al. Highly Reliable Superhydrophobic Protection for Organic Field-Effect Transistors by Fluoroalkylsilane-Coated TiO2 Nanoparticles. *ACS Nano*. **12**, 11062–11069 (2018).
20. Zeng, X. et al. Inspired by Stenocara Beetles: From Water Collection to High-Efficiency Water-in-Oil Emulsion Separation. *ACS Nano*. **11**, 760–769 (2017).
21. Tang, X. & Wang, L. Loss-Free Photo-Manipulation of Droplets by Pyroelectro-Trapping on Superhydrophobic Surfaces. *ACS Nano*. **12**, 8994–9004 (2018).
22. Wong, W. S. Y. et al. Omnidirectional Self-Assembly of Transparent Superoleophobic Nanotextures. *ACS Nano*. **11**, 587–596 (2017).
23. Chen, X. C., Huang, W. P., Ren, K. F. & Ji, J. Self-healing label materials based on photo-cross-linkable polymeric films with dynamic surface structures. *ACS Nano*. **12**, 8686–8696 (2018).
24. Han, K., Heng, L. & Jiang, L. Multiphase Media Antiadhesive Coatings: Hierarchical Self-Assembled Porous Materials Generated Using Breath Figure Patterns. *ACS Nano*. **10**, 11087–11095 (2016).
25. Tian, D. et al. Fast Responsive and Controllable Liquid Transport on a Magnetic Fluid/Nanoarray Composite Interface. *ACS Nano*. **10**, 6220–6226 (2016).
26. Baidya, A. et al. Organic Solvent-Free Fabrication of Durable and Multifunctional Superhydrophobic Paper from Waterborne Fluorinated Cellulose Nanofiber Building Blocks. *ACS Nano*. **11**, 11091–11099 (2017).
27. Xiang, Z. et al. Reduced Graphene Oxide-Reinforced Polymeric Films with Excellent Mechanical Robustness and Rapid and Highly Efficient Healing Properties. *ACS Nano*. **11**, 7134–7141 (2017).
28. Sun, X. & Rykaczewski, K. Suppression of Frost Nucleation Achieved Using the Nanoengineered Integral Humidity Sink Effect. *ACS Nano*. **11**, 906–917 (2017).
29. Lahiri, S. K., Zhang, P., Zhang, C. & Liu, L. Robust Fluorine-Free and Self-Healing Superhydrophobic Coatings by H_3_BO_3_ Incorporation with SiO_2_ -Alkyl-Silane@PDMS on Cotton Fabric. *ACS Appl. Mater. Interfaces*. **11**, 10262–10275 (2019).
30. Li, T. et al. Janus Polyvinylidene Fluoride Membrane with Extremely Opposite Wetting Surfaces via One Single-Step Unidirectional Segregation Strategy. *ACS Appl. Mater. Interfaces*. **10**, 24947–24954 (2018).
31. Wang, M. et al. Fast Strategy to Functional Paper Surfaces. *ACS Appl. Mater. Interfaces*. **11**, 14445–14456 (2019).
32. Ren, T., Yang, M., Wang, K., Zhang, Y. & He, J. CuO Nanoparticles-Containing Highly Transparent and Superhydrophobic Coatings with Extremely Low Bacterial Adhesion and Excellent Bactericidal Property. *ACS Appl. Mater. Interfaces*. **10**, 25717–25725 (2018).
33. Shim, J., Seo, D., Oh, S., Lee, J. & Nam, Y. Condensation Heat-Transfer Performance of Thermally Stable Superhydrophobic Cerium-Oxide Surfaces. *ACS Appl. Mater. Interfaces*. **10**, 31765–31776 (2018).
34. Fang, C. et al. Antireflective Paraboloidal Microlens Film for Boosting Power Conversion Efficiency of Solar Cells. *ACS Appl. Mater. Interfaces*. **10**, 21950–21956 (2018).
35. Li, Y., Li, B., Zhao, X., Tian, N. & Zhang, J. Totally Waterborne, Nonfluorinated, Mechanically Robust, and Self-Healing Superhydrophobic Coatings for Actual Anti-Icing. *ACS Appl. Mater. Interfaces*. **10**, 39391–39399 (2018).
36. Huang, Z. et al. TDI/TiO_2_ Hybrid Networks for Superhydrophobic Coatings with Superior UV Durability and Cation Adsorption Functionality. *ACS Appl. Mater. Interfaces*. **11**, 7488–7497 (2019).
37. Jiang, G., Chen, L., Zhang, S. & Huang, H. Superhydrophobic SiC/CNTs Coatings with Photothermal Deicing and Passive Anti-Icing Properties. *ACS Appl. Mater. Interfaces*. **10**, 36505–36511 (2018).
38. Rowthu, S. & Hoffmann, P. Perfluoropolyether-Impregnated Mesoporous Alumina Composites Overcome the Dewetting-Tribological Properties Trade-Off. *ACS Appl. Mater. Interfaces*. **10**, 10560–10570 (2018).
39. Su, X., Li, H., Lai, X., Chen, Z. & Zeng, X. Highly Stretchable and Conductive Superhydrophobic Coating for Flexible Electronics. *ACS Appl. Mater. Interfaces*. **10**, 10587–10597 (2018).
40. Lee, Y., You, E. A. & Ha, Y. G. Rationally Designed, Multifunctional Self-Assembled Nanoparticles for Covalently Networked, Flexible and Self-Healable Superhydrophobic Composite Films. *ACS Appl. Mater. Interfaces*. **10**, 9823–9831 (2018).
41. Banuprasad, T. N. et al. Fast Transport of Water Droplets over a Thermo-Switchable Surface Using Rewritable Wettability Gradient. *ACS Appl. Mater. Interfaces*. **9**, 28046–28054 (2017).
42. Wang, Y. et al. Multifunctional superhydrophobic surfaces templated from innately microstructured hydrogel matrix. *Nano Lett.* **14**, 4803–4809 (2014).
43. Wang, P., Zhang, D., Sun, S., Li, T. & Sun, Y. Fabrication of slippery lubricant-infused porous surface with high underwater transparency for the control of marine biofouling. *ACS Appl. Mater. Interfaces*. **9**, 972–982 (2017).
44. Emelyanenko, A. M., Boinovich, L. B., Bezdomnikov, A. A., Chulkova, E. V. & Emelyanenko, K. A. Reinforced Superhydrophobic Coating on Silicone Rubber for Longstanding Anti-Icing Performance in Severe Conditions. *ACS Appl. Mater. Interfaces*. **9**, 24210–24219 (2017).
45. Shen, Y. et al. Spraying Fabrication of Durable and Transparent Coatings for Anti-Icing Application: Dynamic Water Repellency, Icing Delay, and Ice Adhesion. *ACS Appl. Mater. Interfaces*. **11**, 3590–3598 (2019).
46. Guo, J. et al. Superhydrophobic and Slippery Lubricant-Infused Flexible Transparent Nanocellulose Films by Photoinduced Thiol-Ene Functionalization. *ACS Appl. Mater. Interfaces*. **8**, 34115–34122 (2016).
47. Zhang, J., Gu, C. & Tu, J. Robust Slippery Coating with Superior Corrosion Resistance and Anti-Icing Performance for AZ31B Mg Alloy Protection. *ACS Appl. Mater. Interfaces*. **9**, 11247–11257 (2017).
48. Bao, B. et al. Patterning liquids on inkjet-imprinted surfaces with highly adhesive superhydrophobicity. *Nanoscale*. **8**, 9556–9562 (2016).
49. Xie, H., Huang, H. X. & Peng, Y. J. Rapid fabrication of bio-inspired nanostructure with hydrophobicity and antireflectivity on polystyrene surface replicating from cicada wings. *Nanoscale*. **9**, 11951–11958 (2017).
50. Li, J. et al. Superhydrophobic meshes that can repel hot water and strong corrosive liquids used for efficient gravity-driven oil/water separation. *Nanoscale*. **8**, 7638–7645 (2016).
51. Iqbal, R., Majhy, B. & Sen, A. K. Facile Fabrication and Characterization of a PDMS-Derived Candle Soot Coated Stable Biocompatible Superhydrophobic and Superhemophobic Surface. *ACS Appl. Mater. Interfaces*. **9**, 31170–31180 (2017).
52. Vüllers, F. et al. Self-Cleaning Microcavity Array for Photovoltaic Modules. *ACS Appl. Mater. Interfaces*. **10**, 2929–2936 (2018).
53. Kollarigowda, R. H., Abraham, S. & Montemagno, C. D. Antifouling Cellulose Hybrid Biomembrane for Effective Oil/Water Separation. *ACS Appl. Mater. Interfaces*. **9**, 29812–29819 (2017).
54. Li, C., Gu, Y. & Zacharia, N. S. Tuning Wet Adhesion of Weak Polyelectrolyte Multilayers. *ACS Appl. Mater. Interfaces*. **10**, 7401–7412 (2018).
55. Li, J. et al. Hydrophobic liquid-infused porous polymer surfaces for antibacterial applications. *ACS Appl. Mater. Interfaces*. **5**, 6704–6711 (2013).
56. Xu, Q. F., Mondal, B. & Lyons, A. M. Fabricating superhydrophobic polymer surfaces with excellent abrasion resistance by a simple lamination templating method. *ACS Appl. Mater. Interfaces*. **3**, 3508–3514 (2011).
57. Rowthu, S., Böhlen, K., Bowen, P. & Hoffmann, P. Surface 3D Micro Free Forms: Multifunctional Microstructured Mesoporous α-Alumina by in Situ Slip Casting Using Excimer Laser Ablated Polycarbonate Molds. *ACS Appl. Mater. Interfaces*. **7**, 24458–24469 (2015).
58. Su, Q., Ma, X., Dong, J., Jiang, C. & Qian, W. A reproducible SERS substrate based on electrostatically assisted aptes-functionalized surface-assembly of gold nanostars. *ACS Appl. Mater. Interfaces*. **3**, 1873–1879 (2011).
59. Li, J. et al. Reactive superhydrophobic surface and its photoinduced disulfide-ene and thiol-ene (Bio)functionalization. *Nano Lett.* **15**, 675–681 (2015).
60. Tuvshindorj, U., Yildirim, A., Ozturk, F. E. & Bayindir, M. Robust cassie state of wetting in transparent superhydrophobic coatings. *ACS Appl. Mater. Interfaces*. **6**, 9680–9688 (2014).
61. Fu, Y., Jin, B., Zhang, Q., Zhan, X. & Chen, F. PH-Induced Switchable Superwettability of Efficient Antibacterial Fabrics for Durable Selective Oil/Water Separation. *ACS Appl. Mater. Interfaces*. **9**, 30161–30170 (2017).
62. Jia, L. C. et al. Robustly Superhydrophobic Conductive Textile for Efficient Electromagnetic Interference Shielding. *ACS Appl. Mater. Interfaces*. **11**, 1680–1688 (2019).
63. Guselnikova, O., Elashnikov, R., Postnikov, P., Svorcik, V. & Lyutakov, O. Smart, Piezo-Responsive Polyvinylidenefluoride/Polymethylmethacrylate Surface with Triggerable Water/Oil Wettability and Adhesion. *ACS Appl. Mater. Interfaces*. **10**, 37461–37469 (2018).
64. Peng, S. et al. Photocatalytically Stable Superhydrophobic and Translucent Coatings Generated from PDMS-Grafted-SiO_2_ /TiO_2_ @PDMS with Multiple Applications. *Langmuir*. **35**, 2760–2771 (2019).
65. Hen, M. et al. Microfluidic Devices Containing ZnO Nanorods with Tunable Surface Chemistry and Wetting-Independent Water Mobility. *Langmuir*. **35**, 3265–3271 (2019).
66. Xiao, Z., Wang, Q., Yao, D., Yu, X. & Zhang, Y. Enhancing the Robustness of Superhydrophobic Coatings via the Addition of Sulfide. *Langmuir*. **35**, 6650–6656 (2019).
67. Wu, B. et al. Molding processed multi-layered and multi-functional nanocomposites with high structural ability, electrical conductivity and durable superhydrophobicity. *Nanoscale*. **10**, 19916–19926 (2018).
68. Li, B. et al. Planting carbon nanotubes onto supramolecular polymer matrices for waterproof non-contact self-healing. *Nanoscale*. **11**, 467–473 (2019).
69. Qiang, S. et al. Wearable solid-state capacitors based on two-dimensional material all-textile heterostructures. *Nanoscale*. **11**, 9912–9919 (2019).
70. Wang, Y. et al. A multifunctional polymeric nanofilm with robust chemical performances for special wettability. *Nanoscale*. **8**, 5153–5161 (2016).
71. Wu, H. et al. Theoretical and Experimental Study of Reversible and Stable Wetting States of a Hierarchically Wrinkled Surface Tuned by Mechanical Strain. *Langmuir*. **35**, 6870–6877 (2019).
72. Park, J. K., Yang, Z. & Kim, S. Black Silicon/Elastomer Composite Surface with Switchable Wettability and Adhesion between Lotus and Rose Petal Effects by Mechanical Strain. *ACS Appl. Mater. Interfaces*. **9**, 33333–33340 (2017).
73. De Haan, L. T. et al. Contactless Control of Local Surface Buckling in Photoaligned Gold/Liquid Crystal Polymer Bilayers. *Langmuir*. **34**, 10543–10549 (2018).
74. Zhu, G. H., Cho, S. H., Zhang, H., Zhao, M. & Zacharia, N. S. Slippery Liquid-Infused Porous Surfaces (SLIPS) Using Layer-by-Layer Polyelectrolyte Assembly in Organic Solvent. *Langmuir*. **34**, 4722–4731 (2018).
75. Zhao, S. et al. Sequentially Reinforced Additive Coating for Transparent and Durable Superhydrophobic Glass. *Langmuir*. **34**, 11316–11324 (2018).
76. Walker, C. et al. Transparent Metasurfaces Counteracting Fogging by Harnessing Sunlight. *Nano Lett.* **19**, 1595–1604 (2019).
77. Chen, F. F. et al. Hydroxyapatite Nanowire-Based All-Weather Flexible Electrically Conductive Paper with Superhydrophobic and Flame-Retardant Properties. *ACS Appl. Mater. Interfaces*. **9**, 39534–39548 (2017).
78. Yu, L., Chen, G. Y., Xu, H. & Liu, X. Substrate-independent, transparent oil-repellent coatings with self-healing and persistent easy-sliding oil repellency. *ACS Nano*. **10**, 1076–1085 (2016).
79. Guo, B. et al. Efficient Flame-Retardant and Smoke-Suppression Properties of Mg-Al-Layered Double-Hydroxide Nanostructures on Wood Substrate. *ACS Appl. Mater. Interfaces*. **9**, 23039–23047 (2017).
80. Xu, W. et al. On-Demand Capture and Release of Organic Droplets Using Surfactant-Doped Polypyrrole Surfaces. *ACS Appl. Mater. Interfaces*. **9**, 23119–23127 (2017).
81. Manabe, K. et al. Controllable Broadband Optical Transparency and Wettability Switching of Temperature-Activated Solid/Liquid-Infused Nanofibrous Membranes. *ACS Nano*. **10**, 9387–9396 (2016).
82. Okada, I. & Shiratori, S. High-transparency, self-standable Gel-SLIPS fabricated by a facile nanoscale phase separation. *ACS Appl. Mater. Interfaces*. **6**, 1502–1508 (2014).
83. Manabe, K., Kyung, K. H. & Shiratori, S. Biocompatible slippery fluid-infused films composed of chitosan and alginate via layer-by-layer self-assembly and their antithrombogenicity. *ACS Appl. Mater. Interfaces*. **7**, 4763–4771 (2015).
84. Xu, W., Song, J., Sun, J., Lu, Y. & Yu, Z. Rapid fabrication of large-area, corrosion-resistant superhydrophobic Mg alloy surfaces. *ACS Appl. Mater. Interfaces*. **3**, 4404–4414 (2011).
85. De Leon, A. C. C., Pernites, R. B. & Advincula, R. C. Superhydrophobic colloidally textured polythiophene film as superior anticorrosion coating. *ACS Appl. Mater. Interfaces*. **4**, 3169–3176 (2012).
86. Wang, J., Kato, K., Blois, A. P. & Wong, T. S. Bioinspired Omniphobic Coatings with a Thermal Self-Repair Function on Industrial Materials. *ACS Appl. Mater. Interfaces*. **8**, 8265–8271 (2016).
87. Wang, B. et al. Methodology for robust superhydrophobic fabrics and sponges from in situ growth of transition metal/metal oxide nanocrystals with thiol modification and their applications in oil/water separation. *ACS Appl. Mater. Interfaces*. **5**, 1827–1839 (2013).
88. Wei, C., Zhang, G., Zhang, Q., Zhan, X. & Chen, F. Silicone Oil-Infused Slippery Surfaces Based on Sol-Gel Process-Induced Nanocomposite Coatings: A Facile Approach to Highly Stable Bioinspired Surface for Biofouling Resistance. *ACS Appl. Mater. Interfaces*. **8**, 34810–34819 (2016).
89. Hsu, C. P., Chang, L. Y., Chiu, C. W., Lee, P. T. C. & Lin, J. J. Facile fabrication of robust superhydrophobic epoxy film with polyamine dispersed carbon nanotubes. *ACS Appl. Mater. Interfaces*. **5**, 538–545 (2013).
90. Ou, J. et al. Tunable water adhesion on titanium oxide surfaces with different surface structures. *ACS Appl. Mater. Interfaces*. **4**, 5737–5741 (2012).
91. Genzer, J. & Efimenko, K. Creating long-lived superhydrophobic polymer surfaces through mechanically assembled monolayers. *Science.* **290**, 2130–2133 (2000).
92. Kang, S. M. et al. One-step modification of superhydrophobic surfaces by a mussel-inspired polymer coating. *Angew. Chem. Int. Ed.* **49**, 9401–9404 (2010).
93. Wu, D. et al. A facile approach for artificial biomimetic surfaces with both superhydrophobicity and iridescence. *Soft Matter*. **6**, 263–267 (2010).
94. Li, X. et al. Periodic parallel array of nanopillars and nanoholes resulting from colloidal stripes patterned by geometrically confined evaporative self-assembly for unique anisotropic wetting. *ACS Appl. Mater. Interfaces*. **6**, 20300–20308 (2014).
95. Masheder, B., Urata, C., Cheng, D. F. & Hozumi, A. Novel transparent zirconium-based hybrid material with multilayered nanostructures: Studies of surface dewettability toward alkane liquids. *ACS Appl. Mater. Interfaces*. **5**, 154–163 (2013).
96. Chang, K. C. et al. Nanocasting technique to prepare lotus-leaf-like superhydrophobic electroactive polyimide as advanced anticorrosive coatings. *ACS Appl. Mater. Interfaces*. **5**, 1460–1467 (2013).
97. Brassard, J. D., Sarkar, D. K. & Perron, J. Synthesis of monodisperse fluorinated silica nanoparticles and their superhydrophobic thin films. *ACS Appl. Mater. Interfaces*. **3**, 3583–3588 (2011).
98. Cheng, Z., Du, M., Fu, K., Zhang, N. & Sun, K. PH-controllable water permeation through a nanostructured copper mesh film. *ACS Appl. Mater. Interfaces*. **4**, 5826–5832 (2012).
99. Jin, H., Tian, X., Ikkala, O. & Ras, R. H. A. Preservation of superhydrophobic and superoleophobic properties upon wear damage. *ACS Appl. Mater. Interfaces*. **5**, 485–488 (2013).
100. Shirtcliffe, N. J., McHale, G., Newton, M. I., Perry, C. C. & Roach, P. Porous materials show superhydrophobic to superhydrophilic switching. *ChemComm*. 3135–3137 (2005).
101. Wu, J., Xia, J., Lei, W. & Wang, B. Superhydrophobic surface based on a coral-like hierarchical structure of ZnO. *PLoS One*. **5**, e14475 (2010).
102. Tadanaga, K., Morinaga, J., Matsuda, A. & Minami, T. Superhydrophobic - Superhydrophilic micropatterning on flowerlike alumina coating film by the sol - Gel method. *Chem. Mater.* **12**, 590–592 (2000).
103. Chen, L. et al. Thermal-responsive hydrogel surface: Tunable wettability and adhesion to oil at the water/solid interface. *Soft Matter*. **6**, 2708–2712 (2010).
104. Barshilia, H. C., Tej, K. R. S., Devi, L. M. & Rajam, K. S. Nanometric multiscale rough Zn-ZnO superhydrophobic thin films: Self-diffusion of zinc and effect of UV irradiation. *J. Appl. Phys.* **108**, 074315 (2010).
105. Rosario, R. et al. Lotus effect amplifies light-induced contact angle switching. *J. Phys. Chem. B*. **108**, 12640–12642 (2004).
106. Taniguchi, T., Kyung, K. H. & Shiratori, S. Layer-by-layer self-assembled thin films of chitin fibers and heparin with anti-thrombus characteristics. *RSC Adv.* **5**, 107488–107496 (2015).
107. Wang, Z., Koratkar, N., Ci, L. & Ajayan, P. M. Combined micro-/nanoscale surface roughness for enhanced hydrophobic stability in carbon nanotube arrays. *Appl. Phys. Lett.* **90**, 143117 (2007).
108. Ferrari, M., Ravera, F. & Liggieri, L. Preparation of a superhydrophobic surface by mixed inorganic-organic coating. *Appl. Phys. Lett.* **88**, 203125 (2006).
109. Ding, B. et al. Conversion of an electrospun nanofibrous cellulose acetate mat from a super-hydrophilic to super-hydrophobic surface. *Nanotechnology*. **17**, 4332–4339 (2006).
110. Chen, F. et al. Table Salt as a Template to Prepare Reusable Porous PVDF–MWCNT Foam for Separation of Immiscible Oils/Organic Solvents and Corrosive Aqueous Solutions. *Adv. Funct. Mater.* **27**, 1702926 (2017).
111. Shang, H. M. et al. Optically transparent superhydrophobic silica-based films. *Thin Solid Films*. **472**, 37–43 (2005).
112. Jiang, W. et al. Photo-switched wettability on an electrostatic self-assembly azobenzene monolayer. *ChemComm*. **28**, 3550–3552 (2005).
113. Li, G. et al. Tunable wettability in surface-modified ZnO-based hierarchical nanostructures. *Appl. Phys. Lett.* **92**, 173104-173104–3 (2008).
114. Liu, K., Zhang, M., Zhai, J., Wang, J. & Jiang, L. Bioinspired construction of Mg-Li alloys surfaces with stable superhydrophobicity and improved corrosion resistance. *Appl. Phys. Lett.* **92**, 183103 (2008).
115. Lee, Y., Lee, W. & Lee, J. K. Fabrication of hierarchical structures on a polymer surface using patterned anodic aluminum oxide as a replication master. *Thin Solid Films*. **516**, 3431–3435 (2008).
116. Wang, B. & Guo, Z. Superhydrophobic copper mesh films with rapid oil/water separation properties by electrochemical deposition inspired from butterfly wing. *Appl. Phys. Lett.* **103**, 063704 (2013).
117. Chen, Y. et al. Transparent superhydrophobic/superhydrophilic coatings for self-cleaning and anti-fogging. *Appl. Phys. Lett.* **101**, 033701 (2012).
118. Barshilia, H. C., Mohan, D. K., Selvakumar, N. & Rajam, K. S. Effect of substrate roughness on the apparent surface free energy of sputter deposited superhydrophobic polytetrafluoroethylene thin films. *Appl. Phys. Lett.* **95**, 033116 (2009).
119. Cai, C. Y., Lin, K. Y. A. & Yang, H. Superhydrophobic anti-ultraviolet films by doctor blade coating. *Appl. Phys. Lett.* **105**, 201913 (2014).
120. Zhai, S. & Zhao, H. Silica-coated metallic nanoparticle-based hierarchical super-hydrophobic surfaces fabricated by spin-coating and inverse nanotransfer printing. *Appl. Phys. Lett.* **114**, 233702 (2019).
121. Neto, A. I., Vasconcelos, N. L., Oliveira, S. M., Ruiz-Molina, D. & Mano, J. F. High-Throughput Topographic, Mechanical, and Biological Screening of Multilayer Films Containing Mussel-Inspired Biopolymers. *Adv. Funct. Mater.* **26**, 2745–2755 (2016).
122. Fukada, K., Taniguchi, T. & Shiratori, S. Viscoelastic and durability analysis of nanostructured composite layers of polyelectrolyte and nanoparticles. *RSC Adv.* **5**, 52837–52843 (2015).
123. Sunny, S., Vogel, N., Howell, C., Vu, T. L. & Aizenberg, J. Lubricant-Infused Nanoparticulate Coatings Assembled by Layer-by-Layer Deposition. *Adv. Funct. Mater.* **24**, 6658–6667 (2014).
124. Yao, T. et al. Fabrication of flexible superhydrophobic films by lift-up soft-lithography and decoration with Ag nanoparticles. *Nanotechnology*. **20**, 065304 (2009).
125. Qing, G., Wang, X., Jiang, L., Fuchs, H. & Sun, T. Saccharide-sensitive wettability switching on a smart polymer surface. *Soft Matter*. **5**, 2759–2765 (2009).
126. Lin, P. C. & Yang, S. Mechanically switchable wetting on wrinkled elastomers with dual-scale roughness. *Soft Matter*. **5**, 1011–1018 (2009).
127. Bayer, I. S., Steele, A., Martorana, P. J., Loth, E. & Miller, L. Superhydrophobic cellulose-based bionanocomposite films from Pickering emulsions. *Appl. Phys. Lett.* **94**, (2009).
128. Lyu, S. & Hwang, W. Facile stamp patterning method for superhydrophilic/superhydrophobic surfaces. *Appl. Phys. Lett.* **107**, 201606 (2015).
129. Zhang, W., Wang, S., Yu, X. & Zhang, Y. Fabrication of non-modified metallic superhydrophobic surfaces with temperature insensitivity and self-healing ability. *Appl. Phys. Lett.* **109**, 043702 (2016).
130. Pogreb, R., Whyman, G., Barayev, R., Bormashenko, E. & Aurbach, D. A reliable method of manufacturing metallic hierarchical superhydrophobic surfaces. *Appl. Phys. Lett.* **94**, 221902 (2009).
131. Wang, G. et al. Conductive and transparent superhydrophobic films on various substrates by in situ deposition. *Appl. Phys. Lett.* **102**, 203703 (2013).
132. Juuti, P. et al. Achieving a slippery, liquid-infused porous surface with anti-icing properties by direct deposition of flame synthesized aerosol nanoparticles on a thermally fragile substrate. *Appl. Phys. Lett.* **110**, 161603 (2017).
133. You, I., Lee, T. G., Nam, Y. S. & Lee, H. Fabrication of a micro-omnifluidic device by omniphilic/omniphobic patterning on nanostructured surfaces. *ACS Nano*. **8**, 9016–9024 (2014).
134. Brabcova, Z., McHale, G., Wells, G. G., Brown, C. V. & Newton, M. I. Electric field induced reversible spreading of droplets into films on lubricant impregnated surfaces. *Appl. Phys. Lett.* **110**, 121603 (2017).
135. Doll, K. et al. Liquid-Infused Structured Titanium Surfaces: Antiadhesive Mechanism to Repel Streptococcus oralis Biofilms. *ACS Appl. Mater. Interfaces*. **11**, 23026–23038 (2019).
136. Chen, C. et al. In Situ Reversible Control between Sliding and Pinning for Diverse Liquids under Ultra-Low Voltage. *ACS Nano*. **13**, 5742–5752 (2019).
137. Jing, X. & Guo, Z. Fabrication of biocompatible super stable lubricant-immobilized slippery surfaces by grafting a polydimethylsiloxane brush: Excellent boiling water resistance, hot liquid repellency and long-term slippery stability. *Nanoscale*. **11**, 8870–8881 (2019).
138. Daniel, D., Mankin, M. N., Belisle, R. A., Wong, T. S. & Aizenberg, J. Lubricant-infused micro/nano-structured surfaces with tunable dynamic omniphobicity at high temperatures. *Appl. Phys. Lett.* **102**, 231603 (2013).
139. Jiao, Y. et al. Pitcher plant-bioinspired bubble slippery surface fabricated by femtosecond laser for buoyancy-driven bubble self-transport and efficient gas capture. *Nanoscale*. **11**, 1370–1378 (2019).
140. Xu, Q. et al. A new kind of transparent and self-cleaning film for solar cells. *Nanoscale*. **8**, 17747–17751 (2016).
141. Sun, Y. & Guo, Z. A scalable, self-healing and hot liquid repelling superamphiphobic spray coating with remarkable mechanochemical robustness for real-life applications. *Nanoscale*. **11**, 13853–13862 (2019).
142. Nguyen-Tri, P. et al. Robust Superhydrophobic Cotton Fibers Prepared by Simple Dip-Coating Approach Using Chemical and Plasma-Etching Pretreatments. *ACS Omega*. **4**, 7829–7837 (2019).
143. Jung, K. K., Jung, Y., Choi, C. J. & Ko, J. S. Highly Reliable Superhydrophobic Surface with Carbon Nanotubes Immobilized on a PDMS/Adhesive Multilayer. *ACS Omega*. **3**, 12956–12966 (2018).
144. Zhang, J. et al. Bio-Inspired Elastic Liquid-Infused Material for On-Demand Underwater Manipulation of Air Bubbles. *ACS Nano*. **13**, 10596–10602 (2019).
145. Jing, X. & Guo, Z. Durable Lubricant-Impregnated Surfaces for Water Collection under Extremely Severe Working Conditions. *ACS Appl. Mater. Interfaces*. **11**, 35949–35958 (2019).
146. Wu, L. et al. Durable and Multifunctional Superhydrophobic Coatings with Excellent Joule Heating and Electromagnetic Interference Shielding Performance for Flexible Sensing Electronics. *ACS Appl. Mater. Interfaces*. **11**, 34338–34347 (2019).
147. Pang, B. et al. 5S Multifunctional Intelligent Coating with Superdurable, Superhydrophobic, Self-Monitoring, Self-Heating, and Self-Healing Properties for Existing Construction Application. *ACS Appl. Mater. Interfaces*. **11**, 29242–29254 (2019).
148. Jamil, M. I., Zhan, X., Chen, F., Cheng, D. & Zhang, Q. Durable and Scalable Candle Soot Icephobic Coating with Nucleation and Fracture Mechanism. *ACS Appl. Mater. Interfaces*. **11**, 31532–31542 (2019).
149. Han, Z. et al. Flexible Self-Cleaning Broadband Antireflective Film Inspired by the Transparent Cicada Wings. *ACS Appl. Mater. Interfaces*. **11**, 17019–17027 (2019).
150. Ezazi, M. et al. Self-Healable Superomniphobic Surfaces for Corrosion Protection. *ACS Appl. Mater. Interfaces*. **11**, 30240–30246 (2019).
151. Qu, M. et al. Multifunctional Superwettable Material with Smart pH Responsiveness for Efficient and Controllable Oil/Water Separation and Emulsified Wastewater Purification. *ACS Appl. Mater. Interfaces*. **11**, 24668–24682 (2019).
152. Li, Q. et al. Superhydrophobic Electrically Conductive Paper for Ultrasensitive Strain Sensor with Excellent Anticorrosion and Self-Cleaning Property. *ACS Appl. Mater. Interfaces*. **11**, 21904–21914 (2019).
153. Wu, Y., Zhou, S., You, B. & Wu, L. Bioinspired Design of Three-Dimensional Ordered Tribrachia-Post Arrays with Re-entrant Geometry for Omniphobic and Slippery Surfaces. *ACS Nano*. **11**, 8265–8272 (2017).
154. Gemici, Z., Shimomura, H., Cohen, R. E. & Rubner, M. F. Hydrothermal treatment of nanoparticle thin films for enhanced mechanical durability. *Langmuir*. **24**, 2168–2177 (2008).
155. Gam-Derouich, S. et al. Highly hydrophilic surfaces from polyglycidol grafts with dual antifouling and specific protein recognition properties. *Langmuir*. **27**, 9285–9294 (2011).
156. Chen, D. et al. Fabricating superhydrophilic wool fabrics. *Langmuir*. **26**, 4675–4679 (2010).
157. Chernyy, S. et al. Superhydrophilic Polyelectrolyte Brush Layers with Imparted Anti-Icing Properties: Effect of Counter ions. *ACS Appl. Mater. Interfaces*. **6**, 6487–6496 (2014).
158. Wang, J. et al. Superhydrophilic Antireflective Periodic Mesoporous Organosilica Coating on Flexible Polyimide Substrate with Strong Abrasion-Resistance. *ACS Appl. Mater. Interfaces*. **9**, 5468–5476 (2017).
159. Cheng, Z., Cheng, K. & Weng, W. SiO_2_/TiO_2_ nanocomposite films on polystyrene for light-induced cell detachment application. *ACS Appl. Mater. Interfaces*. **9**, 2130–2137 (2017).
160. Zhang, L. & Sun, J. Layer-by-layer codeposition of polyelectrolyte complexes and free polyelectrolytes for the fabrication of polymeric coatings. *Macromolecules*. **43**, 2413–2420 (2010).
161. Sett, S. et al. Stable Dropwise Condensation of Ethanol and Hexane on Rationally Designed Ultrascalable Nanostructured Lubricant-Infused Surfaces. *Nano Lett.* **19**, 5287–5296 (2019).
162. Zhai, L., Cebeci, F. C., Cohen, R. E. & Rubner, M. F. Stable superhydrophobic coatings from polyelectrolyte multilayers. *Nano Lett.* **4**, 1349–1353 (2004).
163. Lau, K. K. S. et al. Superhydrophobic Carbon Nanotube Forests. *Nano Lett.* **3**, 1701–1705 (2003).
164. England, M. W., Urata, C., Dunderdale, G. J. & Hozumi, A. Anti-Fogging/Self-Healing Properties of Clay-Containing Transparent Nanocomposite Thin Films. *ACS Appl. Mater. Interfaces*. **8**, 4318–4322 (2016).
165. Xu, L. & He, J. Antifogging and antireflection coatings fabricated by integrating solid and mesoporous silica nanoparticles without any post-treatments. *ACS Appl. Mater. Interfaces*. **4**, 3293–3299 (2012).
166. Fateh, R., Dillert, R. & Bahnemann, D. Self-cleaning properties, mechanical stability, and adhesion strength of transparent photocatalytic TiO2-ZnO coatings on polycarbonate. *ACS Appl. Mater. Interfaces*. **6**, 2270–2278 (2014).
167. Pakdel, A., Bando, Y. & Golberg, D. Plasma-assisted interface engineering of boron nitride nanostructure films. *ACS Nano*. **8**, 10631–10639 (2014).
168. Pegalajar-Jurado, A., Joslin, J. M., Hawker, M. J., Reynolds, M. M. & Fisher, E. R. Creation of hydrophilic nitric oxide releasing polymers via plasma surface modification. *ACS Appl. Mater. Interfaces*. **6**, 12307–12320 (2014).
169. Park, J. H., Oh, J. Y., Han, S. W., Lee, T. Il & Baik, H. K. Low-temperature, solution-processed ZrO2:B Thin film: A bifunctional inorganic/organic interfacial glue for flexible thin-film transistors. *ACS Appl. Mater. Interfaces*. **7**, 4494–4503 (2015).
170. Tiraferri, A., Kang, Y., Giannelis, E. P. & Elimelech, M. Highly hydrophilic thin-film composite forward osmosis membranes functionalized with surface-tailored nanoparticles. *ACS Appl. Mater. Interfaces*. **4**, 5044–5053 (2012).
171. Liang, S. et al. Highly hydrophilic polyvinylidene fluoride (PVDF) ultrafiltration membranes via postfabrication grafting of surface-tailored silica nanoparticles. *ACS Appl. Mater. Interfaces*. **5**, 6694–6703 (2013).
172. Zhang, S. et al. Cupric Phosphate Nanosheets-Wrapped Inorganic Membranes with Superhydrophilic and Outstanding Anticrude Oil-Fouling Property for Oil/Water Separation. *ACS Nano*. **12**, 795–803 (2018).
173. Chang, C. C. et al. Preparation of water-resistant antifog hard coatings on plastic substrate. *Langmuir*. **28**, 17193–17201 (2012).
174. Zhang, W. et al. Facile Design and Fabrication of Superwetting Surfaces with Excellent Wear-Resistance. *ACS Appl. Mater. Interfaces*. **9**, 15776–15784 (2017).
175. Jin, B., He, J., Yao, L., Zhang, Y. & Li, J. Rational Design and Construction of Well-Organized Macro-Mesoporous SiO2/TiO2 Nanostructure toward Robust High-Performance Self-Cleaning Antireflective Thin Films. *ACS Appl. Mater. Interfaces*. **9**, 17466–17475 (2017).
176. Lv, H. et al. Carbon Quantum Dot-Induced MnO2 Nanowire Formation and Construction of a Binder-Free Flexible Membrane with Excellent Superhydrophilicity and Enhanced Supercapacitor Performance. *ACS Appl. Mater. Interfaces*. **9**, 40394–40403 (2017).
177. Soni, R., Kashyap, V., Nagaraju, D. & Kurungot, S. Realizing High Capacitance and Rate Capability in Polyaniline by Enhancing the Electrochemical Surface Area through Induction of Superhydrophilicity. *ACS Appl. Mater. Interfaces*. **10**, 676–686 (2018).
178. Gasparotto, A. et al. Surface Functionalization of Grown-on-Tip ZnO Nanopyramids: From Fabrication to Light-Triggered Applications. *ACS Appl. Mater. Interfaces*. **11**, 15881–15890 (2019).
179. Fu, S. et al. Superhydrophilic, Underwater Directional Oil-Transport Fabrics with a Novel Oil Trapping Function. *ACS Appl. Mater. Interfaces*. **11**, 27402–27409 (2019).
180. Park, S. et al. A Polysaccharide-Based Antibacterial Coating with Improved Durability for Clear Overlay Appliances. *ACS Appl. Mater. Interfaces*. **10**, 17714–17721 (2018).
181. Park, J. A. et al. Quaternized Amphiphilic Block Copolymers/Graphene Oxide and a Poly(vinyl alcohol) Coating Layer on Graphene Oxide/Poly(vinylidene fluoride) Electrospun Nanofibers for Superhydrophilic and Antibacterial Properties. *Sci. Rep.* **9**, 383 (2019).
182. Torun, I. et al. Water Impact Resistant and Antireflective Superhydrophobic Surfaces Fabricated by Spray Coating of Nanoparticles: Interface Engineering via End-Grafted Polymers. *Macromolecules*. **51**, 10011–10020 (2018).
183. Song, J. et al. A superhydrophilic cement-coated mesh: An acid, alkali, and organic reagent-free material for oil/water separation. *Nanoscale*. **10**, 1920–1929 (2018).
184. Lee, H., Alcaraz, M. L., Rubner, M. F. & Cohen, R. E. Zwitter-wettability and antifogging coatings with frost-resisting capabilities. *ACS Nano*. **7**, 2172–2185 (2013).
185. He, Z., Zhuo, Y., Wang, F., He, J. & Zhang, Z. Understanding the role of hollow sub-surface structures in reducing ice adhesion strength. *Soft Matter*. **15**, 2905–2910 (2019).
186. Sekine, T., Tanaka, Y., Sato, C., Tanaka, M. & Hayashi, T. Evaluation of Factors To Determine Platelet Compatibility by Using Self-Assembled Monolayers with a Chemical Gradient. *Langmuir*. **31**, 7100–7105 (2015).
187. Samanta, D., Mehrotra, R., Margulis, K. & Zare, R. N. On-demand electrically controlled drug release from resorbable nanocomposite films. *Nanoscale*. **9**, 16429–16436 (2017).
188. Liang, B., Zhang, G., Zhong, Z., Huang, Y. & Su, Z. Superhydrophilic Anti-Icing Coatings Based on Polyzwitterion Brushes. *Langmuir*. **35**, 1294–1301 (2019).
189. Hsu, W. J. et al. Zeolite-Based Antifogging Coating via Direct Wet Deposition. *Langmuir*. **35**, 2538–2546 (2019).
190. Ye, X. et al. Plasma-Induced, Self-Masking, One-Step Approach to an Ultrabroadband Antireflective and Superhydrophilic Subwavelength Nanostructured Fused Silica Surface. *ACS Appl. Mater. Interfaces*. **10**, 13851–13859 (2018).
191. Fan, Y. J., Pham, M. T. & Huang, C. J. Development of Antimicrobial and Antifouling Universal Coating via Rapid Deposition of Polydopamine and Zwitterionization. *Langmuir*. **35**, 1642–1651 (2019).
192. Maguire-Boyle, S. J. et al. Superhydrophilic Functionalization of Microfiltration Ceramic Membranes Enables Separation of Hydrocarbons from Frac and Produced Water. *Sci. Rep.* **7**, 1–9 (2017).
193. Houshyar, S., Padhye, R., Shanks, R. A. & Nayak, R. Nanodiamond Fabrication of Superhydrophilic Wool Fabrics. *Langmuir*. **35**, 7105–7111 (2019).
194. Li, X., Hu, X. & Cai, T. Construction of Hierarchical Fouling Resistance Surfaces onto Poly(vinylidene fluoride) Membranes for Combating Membrane Biofouling. *Langmuir*. **33**, 4477–4489 (2017).
195. Ye, X. et al. Formation of broadband antireflective and superhydrophilic subwavelength structures on fused silica using one-step self-masking reactive ion etching. *Sci. Rep.* **5**, 1–10 (2015).
196. Xu, Q. C. et al. Superhydrophilicity-assisted preparation of transparent and visible light activated N-doped titania film. *Nanoscale*. **2**, 1122–1127 (2010).
197. Omura, Y., Kyung, K. H., Shiratori, S. & Kim, S. H. Effects of applied voltage and solution ph in fabricating multilayers of weakly charged polyelectrolytes and nanoparticles. *Ind. Eng. Chem. Res.* **53**, 11727–11733 (2014).
198. Fukada, K., Kawamura, N. & Shiratori, S. Trace Material Capture by Controlled Liquid Droplets on a Superhydrophobic/Hydrophilic Surface. *Anal. Chem.* **89**, 10391–10396 (2017).
199. Fukada, K., Nishizawa, S. & Shiratori, S. Antifouling property of highly oleophobic substrates for solar cell surfaces. *J. Appl. Phys.* **115**, 103516 (2014).
200. Ghanbarzadeh, B., Almasi, H. & Entezami, A. A. Physical properties of edible modified starch/carboxymethyl cellulose films. *Innov Food Sci Emerg Technol.* **11**, 697–702 (2010).
